# Cyclodextrin-Based Formulations as a Promising Strategy to Overcome the Blood–Brain Barrier: Historical Overview and Prospects in Glioblastoma Treatment

**DOI:** 10.3390/ph18111626

**Published:** 2025-10-28

**Authors:** Federica De Gaetano, Noemi Totaro, Cinzia Anna Ventura

**Affiliations:** Department of Chemical, Biological, Pharmaceutical and Environmental Sciences, University of Messina, Viale F. Stagno d’Alcontres 31, 98166 Messina, Italy; noemi.totaro@studenti.unime.it (N.T.); caventura@unime.it (C.A.V.)

**Keywords:** glioblastoma, blood-brain barrier, conventional therapy, innovative therapy, cyclodextrins-based formulations

## Abstract

Glioblastoma (GB) is one of the most aggressive and treatment-resistant cancers affecting the central nervous system (CNS), predominantly in adults. Despite significant advancements in this field, GB treatment still relies primarily on conventional approaches, including surgical resection, radiotherapy, and chemotherapy, which, due to its complex pathological characteristics, resistance mechanisms, and restrictive nature of the blood–brain barrier (BBB) and blood–brain tumor barrier (BBTB), remain of limited efficacy. In this context, the development of innovative therapeutic strategies able to overcome these barriers, induce cancer cell death, and improve patient prognosis is crucial. Recently, nanoparticle platforms and focused ultrasounds seem to be promising approaches for cancer treatment. Nanoparticles enable targeting and controlled release, whilst focused ultrasounds enhance tissue permeation, increasing drug accumulation in a specific organ. However, nanoparticles can suffer from synthesis complexity, long-term biocompatibility and accumulation in the body with consequent toxicity, whereas focused ultrasounds require specialized equipment and can potentially cause thermal damage, hemorrhage, or cavitation injury. Cyclodextrins (CYDs) possess good properties and represent a versatile and safer alternative able to improve drug stability, solubility, and bioavailability, and depending on the type, dose, and administration route, can reduce local and systemic toxicity. Thus, CYDs emerge as promising novel excipients in GB treatment. Despite these advantages, CYD complexes suffer from receptor specificity, reducing their potential in precision medicine. By combining CYD complexes with polymeric or lipidic platforms, the advantages of CYD safety and drug solubilization together with their specific targeting can be obtained, thus enhancing selectivity and maximizing efficacy while minimizing recurrence and systemic toxicity. This review provides a comprehensive overview of GB pathology, conventional treatments, and emerging CYD-based strategies aimed at enhancing drug delivery and therapeutic efficacy.

## 1. Introduction

Glioblastoma (GB) is widely recognized as one of the most aggressive and therapeutically challenging malignancies of the central nervous system (CNS), predominantly affecting adults [1]. Clinically, it presents with a range of debilitating symptoms, including headache (≈50%), seizures (20–50%), neurocognitive impairment (30–40%), and focal neurological deficits (10–40%) [2,3]. GB is marked by rapid progression, diffuse infiltration, and an exceptionally poor prognosis. Historically referred to as GB multiforme, the term “multiforme” reflected the tumor’s histopathological heterogeneity. However, this designation was removed in the 2016 update of the World Health Organization (WHO) classification, and the tumor is now simply termed GB [4]. Despite significant advancements in oncology having been reached, particularly in recent decades, GB treatment still relies primarily on conventional approaches including surgical resection, radiotherapy, and chemotherapy [5]. Unfortunately, these approaches offer limited efficacy due to the tumor’s intrinsic resistance mechanisms and complex biology, and the formidable obstacles posed by both the blood–brain barrier (BBB) and the blood–brain tumor barrier (BBTB) [6]. While the BBB restricts the passage of most therapeutic agents into the healthy brain parenchyma, the BBTB—formed through aberrant neovascularization in hypoxic tumor regions—adds an additional layer of complexity. Although structurally more permeable, the BBTB exhibits heterogeneous and selective permeability, which can unpredictably hinder drug distribution within the tumor microenvironment (TME). Together, the BBB and BBTB significantly limit the clinical effectiveness of systemic treatments for GB. In this context, the development of innovative strategies capable of overcoming these barriers and delivering drugs directly to the tumor site is of critical importance [7,8,9,10,11].

Among emerging solutions, cyclodextrins (CYDs) have garnered increasing attention for their ability to enhance drug permeability across the BBB, positioning them as promising excipients in GB therapy [12].

CYDs are cyclic oligosaccharides typically composed of six to eight α-D-glucopyranose units connected via α-1,4-glycosidic bonds. Their distinctive molecular geometry resembles a truncated cone, featuring a hydrophilic exterior and a hydrophobic inner cavity. This structural arrangement allows CYDs to encapsulate a wide range of guest molecules, thereby enhancing their physicochemical properties such as solubility, stability, and permeability [13,14]. In pharmaceutical formulations, CYDs are widely employed as excipients due to their ability to improve the solubility and bioavailability of poorly water-soluble drugs [15]. More recently, their role has expanded into oncology, where CYDs have attracted growing interest for their potential to overcome drug resistance mechanisms and facilitate targeted delivery of therapeutic agents to cancer cells.

In addition to their well-established roles as pharmaceutical excipients, CYDs have recently attracted growing interest in oncology. Their structural versatility and ability to interact with biological membranes and macromolecules have positioned them as promising agents in chemotherapy. Notably, CYDs contribute to overcoming drug resistance by enhancing cellular uptake and enabling more precise delivery of therapeutic compounds to malignant cells [16]. Moreover, CYDs exhibit the distinctive ability to influence the function of efflux transporters located on the BBB, which can facilitate improved drug delivery to the CNS [12]. This property is extremely beneficial in GB therapy, where effective drug passage through the BBB is a critical challenge.

Although CYDs offer significant promise, their application in GB therapy is still evolving. One of the most promising clinical applications of CYD-based formulations in GB therapy is represented by MTX110, a water-soluble form of panobinostat obtained through complexation with hydroxypropyl-β-CYD (HP-β-CD) [17,18,19]. This strategy enables convection-enhanced delivery (CED), allowing direct infusion into the tumor site and bypassing the blood–brain barrier. Notably, the currently available oral formulation of panobinostat lactate (Farydak^®^) is not suitable for the treatment of brain cancers due to poor blood–brain barrier penetration and inadequate drug concentrations in the brain. The MAGIC-G1 Phase I clinical trial (NCT05324501) evaluated MTX110 in patients with recurrent GB, demonstrating encouraging survival outcomes and good tolerability. The use of HP-β-CD not only improves the solubility of panobinostat but also facilitates its local administration at potentially therapeutic concentrations, highlighting the translational potential of CYD-based systems in neuro-oncology.

To maximize the effectiveness of CYDs, ongoing research focuses on refining the design of CYD-based drug delivery systems (DDSs). The goal is to achieve precise targeting of GB cells while reducing adverse effects [20,21]. This is often pursued through the incorporation of specific targeting molecules or the development of nanoparticle-based formulations to enhance selective drug delivery. In addition, investigating the integration of CYDs with standard therapies or novel treatment modalities, such as immunotherapy, presents a promising strategy to address therapy resistance and enhance patient outcomes [22].

Previous reviews have explored CYD-based drug delivery systems in different contexts. Xing et al. [23] provided a comprehensive overview of these systems in neurodegenerative disorders, such as Alzheimer’s and Parkinson’s disease. Their work emphasized the structural versatility of CDs and their ability to interact with biological barriers but did not address the specific challenges associated with GB or the therapeutic strategies required to overcome its highly heterogeneous and invasive nature.

Similarly, Păduraru et al. [24] reviewed the potential of CYD-based drug delivery systems for cancer therapy, highlighting their chemical properties, biocompatibility, and applications across various tumor types. However, this review did not focus on the unique anatomical and physiological constraints of the BBB nor on the translational hurdles in GB treatment.

In contrast, the present review specifically examines CYD-based formulations as a strategy to cross the BBB and BBTB in GB therapy, integrating historical developments, mechanistic insights, and future clinical perspectives. To the best of the authors’ knowledge, no comprehensive review currently exists that combines these aspects in such a challenging setting. This review aims to fill that gap by providing (i) an overview of GB pathology and the limitations of conventional and emerging therapies; (ii) a critical analysis of CD-based strategies to enhance BBB and BBTB permeability and drug delivery; and (iii) future perspectives on their clinical applicability. By focusing on the intersection of BBB biology, GB therapy, and CYD-based nanotechnology, this work highlights a unique and underexplored niche in the literature.

## 2. Classification of GB

### 2.1. World Health Organization Classification

**Figure 1:**
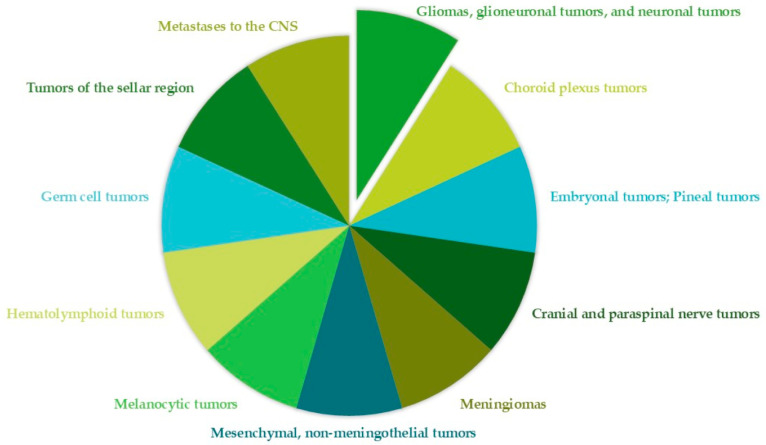
World Health Organization *Classification of major Tumors of the CNS*, fifth edition.

The fifth edition of the *WHO Classification of Tumors of the CNS*, published in 2021, classifies brain tumors into 12 main categories [25], summarized in Figure 1.

Among these, with a median survival of approximately 15 months, GB is still an incurable condition. Five years after diagnosis, just 5.5% of patients survive [26]. Tumors affecting the CNS form a heterogeneous group of neoplasms, classified into two main categories: primary tumors, which account for 90% of cases and arise de novo in the brain, and secondary tumors (or metastases), which make up the remaining 10% and originate from a pre-existing lower-grade tumor, known as a primitive tumor [27]. The pathophysiology of brain metastases is a complex, multistage process driven by molecular pathways. Cancer cells must undergo adaptive changes, proliferate, and migrate from the primary organ to the CNS, where they can remain dormant for varying periods before becoming invasive and spreading [28].

GB is now classified as a CNS WHO grade 4 astrocytic tumor, defined by isocitrate dehydrogenase (IDH)-wildtype and specific histone H3-wildtype. Diagnosis requires at least one of the following molecular alterations: telomerase reverse transcriptase (TERT) promoter mutation, epidermal growth factor receptor (EGFR) gene amplification, or combined whole chromosome 7 gain and whole chromosome 10 loss (+7/−10). These molecular criteria take precedence over histological features such as necrosis or microvascular proliferation, emphasizing the tumor’s aggressive nature [29,30].

A key prognostic factor in GB is cyclin-dependent kinase inhibitor 2A/B (CDKN2A/B) homozygous deletion, which is associated with worse outcomes [31]. Additional molecular markers, such as EGFR amplification and TERT promoter mutations, drive oncogenic pathways that promote uncontrolled proliferation and resistance to senescence [29].

Unlike secondary gliomas, which frequently harbor IDH mutations and have better prognosis, GBs are typically IDH-wildtype. The absence of IDH mutations correlates with a more aggressive clinical course, as IDH-mutant gliomas exhibit a hypermethylated phenotype (G-CIMP) linked to epigenetic dysregulation [32].

The 2021 classification also introduced “Not Otherwise Specified” (NOS) and “Not Elsewhere Classified” (NEC) categories for diagnostically ambiguous cases. NOS applies when molecular data are insufficient, while NEC refers to tumors with atypical features that do not fit existing criteria. These categories facilitate systematic classification and research into rare GB variants [33].

### 2.2. A Biomarker-Based Classification

**Figure 2:**
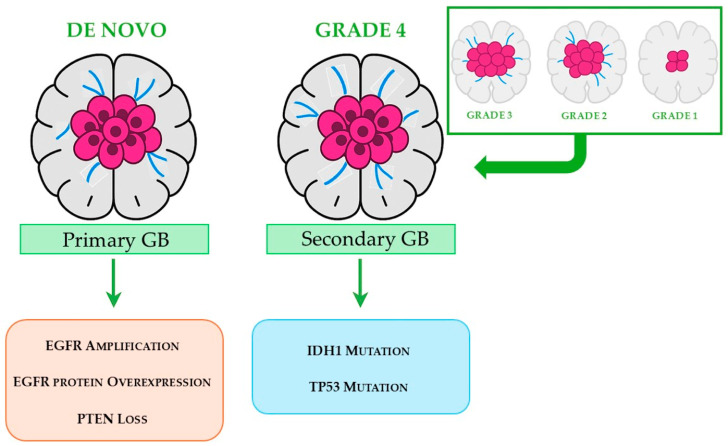
Schematic illustration of primary and secondary GB, including the major molecular biomarkers.

GM is clinically classified into two main categories [34,35], as schematically illustrated in Figure 2. Primary GB, which accounts for approximately 90% of cases, arises de novo and predominantly affects elderly patients. This subtype is characterized by distinct molecular alterations, including amplification of the EGFR gene, overexpression of the EGFR protein, and loss of the tumor suppressor gene phosphatase and tensin homolog (PTEN). Secondary GB accounts for approximately 10% of all cases, usually starts from a lower-grade glioma, and predominantly affects younger patients. Compared to the primary form, it is associated with a more favorable prognosis and is typically characterized by mutations in the isocitrate dehydrogenase 1 (IDH1) gene and tumor protein p53 (TP53) gene.

### 2.3. Molecular Subtypes

GB exhibits profound molecular heterogeneity. The most widely adopted framework is the four-subtype classification proposed by Verhaak et al. (2010), based on integrated genomic analysis of The Cancer Genome Atlas (TCGA) data [36,37,38], listed below:

- Proneural subtype characterized by alterations in Platelet-Derived Growth Factor Receptor Alpha (PDGFRA), IDH1 mutations, and expression of oligodendrocytic lineage markers. This subtype is more common in younger patients and is associated with a relatively better prognosis. Nonetheless, the proneural subtype did not exhibit a markedly different response to chemotherapy and radiotherapy compared to the other molecular subtypes [39].
- Neural subtype exhibiting gene expression profiles that closely resemble those of normal brain tissue and generally showing greater sensitivity to radiation and chemotherapy. GB expressing neural markers such as Synaptotagmin 1, Solute carrier family 12 member 5, Gamma-aminobutyric acid type A receptor alpha1, and Neurofilament light polypeptide are classified within this subtype [39].
- Classical subtype characterized by distinct genomic alterations, including amplification of chromosome 7, loss of chromosome 10, inactivation of the RB (Retinoblastoma-associated protein) pathway, and focal homozygous deletion at 9p21.3. Additionally, this subtype shows high expression of components from the Sonic Hedgehog pathway, the Notch signaling pathway, and the neural precursor/stem cell marker NES. Notably, patients with the classical subtype tend to experience significantly reduced mortality when treated with aggressive radiotherapy and chemotherapy [39].
- Mesenchymal subtype defined by pronounced necrosis and inflammatory activity, along with elevated expression of genes involved in angiogenesis and extracellular matrix remodeling. It frequently presents deletions in key tumor suppressor genes such as TP53, PTEN, and Neurofibromatosis type 1 (NF1), and shows strong activation of the Tumor Necrosis Factor (TNF) superfamily and NF-κB signaling pathways. Despite showing some responsiveness to intensive radiotherapy and chemotherapy, this subtype is associated with the poorest overall prognosis among GB classifications [39].

These subtypes exhibit plasticity and can evolve over time, contributing to therapeutic resistance.

### 2.4. Tumor Microenvironment (TME)

The tumor microenvironment (TME) of GM is a highly dynamic and heterogeneous niche that plays a pivotal role in tumor progression, therapeutic resistance, and recurrence [40]. It comprises a complex network of cellular and non-cellular components, including glioma stem cells, immune cells (such as microglia, macrophages, and lymphocytes), astrocytes, neurons, endothelial cells, and extracellular matrix (ECM) elements [41]. GBM cells actively remodel the TME through direct cell–cell interactions and paracrine signaling, promoting immunosuppression, angiogenesis, and invasion [42]. Notably, the TME contributes to the failure of conventional therapies by fostering adaptive resistance mechanisms and supporting tumor regrowth post treatment [43]. Understanding the intricate crosstalk between GBM cells and their microenvironment is essential for developing more effective therapeutic strategies, including immunomodulatory and ECM-targeted approaches.

## 3. Etiology of GB

The etiology of GB is not yet fully understood and, for this reason, remains an area of active research [44]. Although environmental and lifestyle factors have not been identified as major causes of GB, studies suggest that exposure to ionizing radiation, such as that used in the treatment of other cancers or in high-exposure environments, may increase the risk of developing GB. According to a study, patients with a history of previous therapeutic irradiation had a high rate (17%) [45]. Specifically, radiotherapy is associated with the development of secondary GB in approximately 2.5% of cases [46]. Additionally, some rare genetic conditions, such as neurofibromatosis types 1 and 2, Li-Fraumeni syndrome, and Turcot syndrome, may also increase the risk of developing brain tumors, including GB [47]. There is also evidence suggesting a correlation between certain viral infections and GB. For instance, cytomegalovirus (CMV) has been detected in a substantial number of GB cases, implying that this and potentially other viral infections may play a role in the tumor’s etiology [48]. There is insufficient evidence linking GB to lifestyle variables such as using mobile phones, smoking, diet, drug use, electromagnetic field, severe head injury, pesticide exposure, or being exposed to N-nitroso chemicals [49,50,51]. However, a study by Baglietto et al. has shown that there is a possible correlation between alcohol consumption and the risk of GB [52].

## 4. Epidemiology of GB

The median age of adults diagnosed with GB is 64 years old. As people age, the incidence rises, reaching a high between the ages of 75 and 84, and then falling after 85 [53]. The annual incidence of GB is around 35 per million individuals, with a higher incidence of GB in men as compared to women (a ratio of 1.6:1) [3,54,55]. In addition, as reported, women have a higher incidence in the right temporal lobe, whereas men have a higher incidence in the left temporal lobe [56,57]. GB only accounts for about 3% of all brain and CNS cancers recorded in children aged 0 to 19 [58]. People who have experienced or are now experiencing allergies, asthma, or atopic disorders (e.g., psoriasis, eczema) have a 40% lower risk of acquiring gliomas. The activation of immunological surveillance mechanisms, which are mostly mediated by IgE antibodies, may be the cause of this [51].

The genetic component is crucial since 5–10% of gliomas occur in familial clusters, and first-degree relatives of glioma patients have a twofold greater chance of developing a brain tumor [59]. According to certain research, black people are less likely to develop GB than white people, who are at the highest risk of developing the disease [60].

This review provides a detailed overview of current treatments for GB and aims to examine CYD-based formulations as a potential strategy for overcoming the BBB, with a particular focus on one of the most aggressive brain tumors known.

## 5. Blood–Brain Barrier (BBB) in Brain Cancer

The BBB is composed of microvascular endothelial cells that communicate with brain cells like astrocytes and pericytes while separating blood from the brain interstitial fluid. Tight and adherent junctions between adjacent endothelial cells reinforce the BBB, resulting in such a narrow pore diameter (1.4–1.8 nm) that only a few tiny particles can get through. Actually, nearly all biological macromolecular agents (such as growth factors and antibodies) and around 98% of small molecules are prevented from crossing the BBB, which significantly impairs the delivery of drugs to the brain [61]. The BBB protects invasive tumor cells from therapeutic agents, causing treatment resistance and recurrence [62]. Only 20% of small molecules (molecular weight below 400–600 kDa) and no big therapeutic drugs penetrate the BBB to reach tumor cells at a therapeutic dose level [1,63,64].

The ABC transporter protein family, which exports foreign molecules out of cells and eliminates a range of compounds, including medicines, is one of the BBB’s essential components [65].

**Figure 3:**
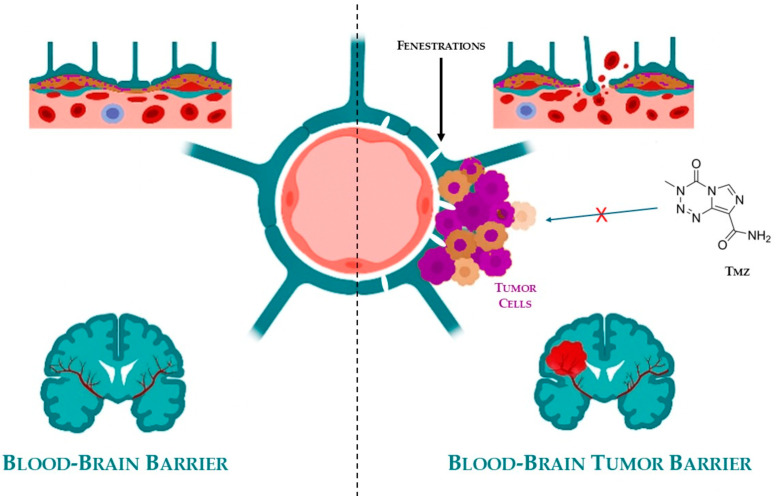
Schematic illustration of the intact blood-brain barrier (BBB, left) and the altered blood-brain tumor barrier (BBTB, right). The BBB is represented with tightly packed endothelial cells (petrol blue) without fenestrations, ensuring restricted permeability. In contrast, the BBTB shows disrupted endothelial structure with fenestrations (highlighted in light gaps) and infiltrating tumor cells (fuchsia), which hinder uniform temozolomide penetration (TMZ, petrol blue arrow). These changes lead to subtherapeutic drug concentrations in critical tumor regions and reduced treatment efficacy (red X).

In addition to the well-established BBB, brain tumors develop a secondary protective layer known as the blood–brain tumor barrier (BBTB), shown in Figure 3.

This barrier arises in response to hypoxic conditions within the TME, which stimulates the overexpression of angiogenic mediators such as vascular endothelial growth factor (VEGF). The result is aberrant neovascularization, characterized by the formation of disorganized and leaky blood vessels that differ significantly from normal cerebral vasculature.

While these newly formed vessels facilitate the delivery of oxygen and nutrients to the tumor—thereby promoting its growth and invasiveness—they paradoxically act as a selective barrier, impeding the penetration of many chemotherapeutic agents. The structural heterogeneity and irregular permeability of the BBTB make drug delivery unpredictable and often ineffective [66,67,68].

Together, the BBB and BBTB significantly limit the clinical efficacy of most anticancer drugs in treating brain tumors. Overcoming these barriers is a critical challenge in neuro-oncology and has prompted the development of novel drug delivery systems, including nanocarriers, CYD-based formulations, and temporary barrier disruption techniques, aimed at enhancing therapeutic access to the tumor site [69,70,71,72]. No systematic review addressing CYD-based formulations in the treatment of brain tumors has been reported in the literature, to the best of the authors’ knowledge.

## 6. State of the Art of Treatment Options

GB remains one of the most aggressive and therapeutically challenging tumors in neuro-oncology. Its highly infiltrative nature, rapid progression, and resistance to conventional therapies contribute to poor prognosis and high recurrence rates. Furthermore, its difficulty crossing the BBTB significantly limits the effectiveness of conventional treatments. The standard of care for GB is based on a multimodal approach that integrates surgical resection [73], radiotherapy [74], and pharmacotherapy [75]. However, due to the tumor’s complexity and its ability to evade therapeutic interventions, additional strategies have emerged to improve clinical outcomes and patient quality of life.

In recent years, the therapeutic landscape has expanded to include innovative modalities such as Tumor Treatment Fields (TTFs), advanced drug delivery systems, and comprehensive supportive care. These approaches aim not only to reduce tumor burden but also to modulate the TME, manage symptoms, and address the multifaceted needs of GB patients.

This section provides a detailed overview of the current treatment options for GB, highlighting their mechanisms, clinical benefits, and limitations. A summary table is included at the end to facilitate comparison and offer a concise reference for clinicians and researchers (Table 1).

### 6.1. Surgical Resection

Surgical resection represents the first and most crucial step in GB management. The benefits of this approach are diverse: in addition to reducing the tumor burden, which remains the primary objective, tumor resection significantly improves clinical symptoms by alleviating intracranial pressure and compression-related effects. Moreover, it enhances the efficacy of subsequent treatments by reducing the number of residual tumor cells [76]. Surgery also plays a pivotal role in confirming the GB diagnosis and identifying specific genetic mutations, which are essential for tailoring an appropriate treatment plan. However, surgical resection is not always feasible, particularly when the tumor is located in deep or functionally critical areas of the brain [77].

Complete removal of tumor cells, which is strongly associated with improved prognosis, is rarely achievable due to the highly infiltrative nature of GB. GB cells extend several centimeters beyond the visible tumor boundary, making total resection impossible and leading to frequent recurrences. Even in cases where no radiologically detectable residual tumor remains, recurrence occurs in approximately 80% of cases near the surgical cavity [78,79].

Given the critical importance of this initial treatment step, neurosurgeons employ advanced techniques to maximize the extent of resection while preserving essential brain areas [80]. One such approach is fluorescence-guided surgery (FGS), which utilizes fluorescent tracers to enhance tumor boundary detection [81,82]. Among the most commonly used agents are 5-aminolevulinic acid (5-ALA) and sodium fluorescein (SF), both of which selectively accumulate in GB cells and fluoresce under specific wavelengths, providing real-time visualization of malignant tissue. Notably, no significant differences have been observed in the effectiveness of these two agents [83].

Another valuable strategy involves the integration of intraoperative imaging modalities to improve surgical precision. Intraoperative magnetic resonance imaging (iMRI) provides real-time visualization of the tumor during surgery, offering surgeons more detailed information on tumor localization and extent [80,84]. Similarly, intraoperative ultrasound (iUS) serves as a complementary imaging tool to enhance surgical accuracy. iUS provides real-time, high-resolution imaging that aids in differentiating tumor tissue from normal brain structures, making it particularly useful in identifying residual tumor tissue intraoperatively. Its portability and ease of use make it an accessible adjunct to GB surgery [85].

Further advancements in intraoperative imaging include Raman spectroscopy, a non-invasive optical technique that enables real-time molecular characterization of tissue. By analyzing the vibrational spectra of molecules within the brain tissue, Raman spectroscopy can differentiate between normal and malignant cells, thus improving tumor delineation [86]. This technique has the potential to minimize residual tumor burden and enhance surgical outcomes [87,88].

Confocal laser endomicroscopy (CLE) represents another promising approach for intraoperative tumor identification. By providing real-time, high-resolution microscopic images of brain tissue, CLE allows neurosurgeons to assess cellular morphology at a microscopic level during surgery. This technique enhances the ability to identify infiltrative tumor margins, offering a more precise resection while minimizing damage to surrounding healthy brain tissue [89].

Despite advancements in surgical techniques, the infiltrative nature of GB necessitates the combination of surgery with adjuvant therapies to target residual tumor cells.

### 6.2. Radiotherapy

Since the 1970s, the combination of surgery and radiotherapy has been the cornerstone of treatment for malignant gliomas, including GB [90]. Radiotherapy is used post-surgery to target any remaining cancer cells that were not removed during the operation, thereby reducing the risk of recurrence. Additionally, in cases of advanced or inoperable GB, radiotherapy can be used palliatively to alleviate symptoms and enhance the patient’s quality of life.

Given its ability to target malignant cells while preserving surrounding healthy tissue, radiation therapy plays a significant role in the management of high-grade gliomas. It has been observed that without radiotherapy, only a few patients with malignant glioma survive beyond six months. With the addition of radiotherapy, median survival for GB extends to about 12 months [91]. This extension in survival is a significant improvement, highlighting the critical role of radiotherapy in GB management.

The current standard of care for GB established by the Stupp protocol [92] involves maximal safe surgical resection followed by radiotherapy and concomitant and adjuvant chemotherapy with TMZ. Radiotherapy is administered as fractionated external beam radiation, delivering a total dose of 60 Gy in 30 fractions over six weeks, combined with daily TMZ (75 mg/m^2^), followed by six cycles of adjuvant TMZ (150–200 mg/m^2^ for 5 days every 28 days). This regimen is typically initiated at the earliest feasible time following surgical intervention, ensuring sufficient postoperative healing, which generally occurs within 4 to 6 weeks [93,94]. The goal of this fractionated approach is to maximize the cytotoxic effects on neoplastic cells while minimizing collateral damage to adjacent healthy tissues [95,96,97].

While this regimen has significantly improved median survival, from 9 months with radiotherapy alone to approximately 14–16 months with the full protocol, it remains palliative rather than curative. Radiotherapy, although essential, is associated with several adverse effects, including fatigue, cerebral edema, alopecia, cutaneous reactions, and cognitive decline due to neuronal damage and radiation necrosis [98,99]. Additionally, some tumors develop radio-resistance, reducing the efficacy of treatment over time [100].

Despite aggressive multimodal therapy, GBM is characterized by a high rate of recurrence. Most patients experience tumor regrowth within 6 to 12 months after initial treatment, largely due to the infiltrative nature of the tumor and the persistence of therapy-resistant cells. This recurrence contributes to the poor prognosis, with a 5-year survival rate below 10%. These limitations underscore the urgent need for innovative therapeutic strategies that can overcome resistance mechanisms and improve long-term outcomes [101].

Although advanced radiotherapeutic techniques such as stereotactic fractionated radiotherapy, the incorporation of radiosensitizers, metabolic radiotherapy, and radioimmunotherapy have been developed, these approaches have not consistently demonstrated substantial clinical benefits when compared to the standard radiotherapy regimen [91].

### 6.3. Pharmacotherapy

In the context of GB management, chemotherapy is predominantly employed as an adjuvant therapy to target residual tumor cells that remain after surgical resection and radiotherapy. This approach aims to prolong survival, delay recurrence, and improve overall outcomes. Chemotherapy and/or radiotherapy are used as primary treatment options only when other therapeutic strategies are not feasible (e.g., in patients who are not candidates for surgery due to the tumor’s location) [102].

The standard treatment protocol for GB consists of concomitant radiotherapy and chemotherapy (RTCT) with temozolomide (TMZ). During RTCT, TMZ is administered at a dose of 75 mg/m^2^/day. Following the completion of RTCT, adjuvant chemotherapy with TMZ continues at a dose of 200 mg/m^2^/day for 5 days every 28 days, for a maximum of 12 cycles. This regimen is also employed in cases of recurrent GBM, i.e., tumors that have relapsed [103].

TMZ, a small alkylating agent capable of crossing the BBB to reach the CNS, has been a cornerstone of GB treatment since its approval in 1999 [104]. Its efficacy was established in the pivotal European Organization for Research and Treatment of Cancer/National Cancer Institute (EORTC/NCI) randomized study by Stupp et al. in 2005 [92], which demonstrated that the addition of TMZ to radiotherapy significantly improved survival compared to radiotherapy alone [105]. TMZ exhibits almost 100% bioavailability and a strong penetration capability into all tissues, including neurological tissue. It reaches peak plasma concentration 20 min after oral administration, indicating rapid intestinal absorption. Additional pharmacokinetic properties include modest protein binding (10–20%) and a plasma half-life of 74–110 min. Approximately 10–15% of TMZ is excreted in the urine, with the majority being eliminated via the hepatobiliary system [106].

The active metabolite, 5-(3-methyl-1-triazen-1-yl)imidazole-4-carboxamide (MTIC), is generated through the spontaneous hydrolysis of TMZ [107]. However, MTIC’s intrinsic characteristics hinder its effective interaction with tumor cell membranes, limiting its ability to infiltrate target cells. This may partially explain the lower-than-expected tumoricidal efficacy of TMZ. The drug exerts its cytotoxic effect by methylating DNA at specific sites: N7-guanine (N7-MeG; 70%), N3-adenine (N3-MeA; 9%), and O6-guanine (O6-MeG; 6%). The critical cytotoxic mechanism is attributed to O6 guanine methylation, which is considered the lethal step in its mechanism of action [108]. Unfortunately, tumor cells exhibit resistance to TMZ through various defense mechanisms [109], including their ability to remove methyl groups from DNA or to alter the mismatch repair system. Beyond these intrinsic mechanisms, one of the most critical factors limiting the efficacy of TMZ and other chemotherapeutic agents is the presence of the BBB, a highly selective and tightly regulated vascular interface that protects the brain from potentially harmful substances. Although essential for maintaining CNS homeostasis, the BBB represents a major challenge in the treatment of brain tumors such as GB, as it limits the passage of most pharmacological agents, including antibiotics, antineoplastic drugs, and neuropeptides [110]. This barrier not only impairs the uniform distribution of TMZ but also contributes to subtherapeutic drug concentrations in critical tumor regions, ultimately reducing treatment efficacy and promoting recurrence (Figure 3, Section 5). Together, the BBB and BBTB represent formidable obstacles to the successful delivery of therapeutic compounds, underscoring the urgent need for innovative strategies to enhance CNS drug bioavailability and achieve effective tumor targeting [111].

TMZ is administered to patients until tumor progression occurs, intolerable toxicity manifests, and/or the patient refuses further treatment [112].

In cases of tumor progression—meaning the patient experiences recurrence either during or after TMZ treatment—several therapeutic options can be considered:

- Procarbazine, lomustine, and vincristine regimen (PCV): Procarbazine is a DNA-alkylating agent, lomustine is a nitrosourea compound capable of crossing the BBB, and vincristine is a vinca alkaloid that disrupts microtubule formation, collectively exerting cytotoxic effects on tumor cells [113].
- TMZ rechallenge: Retreatment with TMZ may be an option in select patients [114,115].
- Repeat surgery: If the location and size of the recurrent tumor allow for safe resection, surgical intervention can be reconsidered [116].
- Enrollment in a clinical trial: Patients may benefit from investigational therapies being tested in ongoing clinical trials [117].

Additionally, carmustine, a nitrosourea alkylating agent, can be employed either as an alternative to TMZ or upon tumor progression following TMZ therapy. It can be administered via intravenous injection or through wafer implantation [118,119]. To mitigate the systemic toxicity associated with intravenous carmustine, biodegradable carmustine-impregnated wafers (Gliadel^®^ wafers) were developed by Brem et al. [120]. These wafers are placed directly onto the walls of the resection cavity during surgery, allowing for localized chemotherapy delivery.

To enhance therapeutic efficacy, combination therapies incorporating agents such as bevacizumab [121], irinotecan [122], etoposide [123], erlotinib [124], and carboplatin [125] have also been explored.

### 6.4. Targeting Tyrosine Kinases and the Tumor Microenvironment in GB

It is also worth noting that, in addition to traditional chemotherapy, in addition to conventional chemotherapy, several targeted therapies are being investigated to improve outcomes in GB. Among these, tyrosine kinase inhibitors (TKIs) have gained attention due to their ability to interfere with key signaling pathways involved in tumor growth, proliferation, and resistance to apoptosis. Tyrosine kinase inhibitors (TKIs) are being explored as potential therapeutic agents for GB. Genome-wide studies have revealed that kinase mutations play critical roles in tumor cell growth, proliferation, invasiveness, and resistance to apoptosis, making them attractive therapeutic targets [126,127,128]. The most commonly mutated tyrosine kinases (TKs) in GB include the EGFR, VEGF, and platelet-derived growth factor receptor-α (PDGFR-α) [129,130]. This section provides an overview of the most relevant TKIs and anti-angiogenic agents currently under investigation or in clinical use for GB. For clarity, a summary table has been added at the end of the section to consolidate key information regarding drug names, mechanisms, efficacy, and side effects.

#### 6.4.1. EGFR-Targeting TKIs

Gefitinib, Erlotinib, Dacomitinib, and Osimertinib are TKIs targeting the EGFR, frequently mutated in non-small cell lung cancer (NSCLC) [131]. Gefitinib and Erlotinib are first-generation reversible TKIs that inhibit EGFR by competing with ATP at its binding site [132]. Dacomitinib is a second-generation irreversible pan-HER inhibitor that provides broader inhibition but is associated with increased toxicity [133]. Osimertinib, a third-generation irreversible TKI, selectively targets both sensitizing EGFR mutations and the T790M resistance mutation, and demonstrates superior efficacy and CNS penetration [134]. Common adverse effects include rash, diarrhea, and paronychia, with Dacomitinib and Osimertinib showing higher rates of dose-limiting toxicities such as interstitial lung disease and cardiotoxicity [135,136].

However, clinical trials revealed limited efficacy in GB, primarily due to poor BBB penetration and the emergence of resistance mechanisms.

#### 6.4.2. TKIs with Enhanced CNS Bioavailability

To overcome the limitations of first-generation EGFR inhibitors, research has shifted toward TKIs with improved CNS penetration. Compounds such as AZD3759, epitinib, and WSD0922 are currently under preclinical and clinical investigation [137]. These agents are designed to achieve higher drug concentrations within the brain, potentially enhancing therapeutic efficacy in GB.

#### 6.4.3. Dacomitinib in EGFR-Mutant GB

Among EGFR-targeting TKIs, dacomitinib has been evaluated in a multicenter, open-label, phase II clinical trial (GEINO-11, NCT01520870) involving patients with recurrent GB harboring EGFR amplification and/or EGFRvIII mutations [138,139]. The study enrolled 49 patients divided into two cohorts: one with EGFR amplification alone, and the other with both EGFR amplification and EGFRvIII mutation.

Dacomitinib was administered orally at 45 mg/day until disease progression or unacceptable toxicity. The primary endpoint, progression-free survival at 6 months (PFS6), was achieved in 10.6% of patients overall (13.3% in Cohort A and 5.9% in Cohort B). The median progression-free survival was 2.7 months, and median overall survival was 7.4 months. Notably, four patients remained progression-free at 6 months, and three patients at 12 months, with one complete response and two partial responses observed. Additionally, stable disease was reported in 24.5% of patients.

Although the overall activity of dacomitinib was limited, these individual durable responses suggest that molecularly selected subgroups of GB patients may benefit from this therapy. Further studies are warranted to identify predictive biomarkers and optimize patient selection.

#### 6.4.4. Regorafenib: A Multi-Kinase Inhibitor

Regorafenib is a multi-kinase inhibitor that targets VEGFR-2 and several other kinases involved in tumor angiogenesis and proliferation. It is currently the most commonly used TKI in clinical practice for recurrent GB. Clinical studies have shown that regorafenib may improve survival compared to lomustine, supporting its use in this setting [140].

#### 6.4.5. Bevacizumab in GB Management

Another therapeutic agent worth mentioning is bevacizumab, a humanized monoclonal antibody that targets VEGF. Although not a TKI, bevacizumab plays a crucial role in GB management, particularly in recurrent cases. Inhibiting the formation of new blood vessels that supply the tumor deprives the tumor of nutrients, thereby slowing its growth [141]. While bevacizumab has demonstrated efficacy in reducing tumor size and delaying disease progression, its impact on overall survival (OS) has been modest [142,143]. Furthermore, like other anti-angiogenic therapies, resistance to bevacizumab may develop over time [144], and its use can be associated with significant side effects, such as increased risk of hemorrhage and thromboembolic events [145].

#### 6.4.6. Bevacizumab for Radionecrosis

Bevacizumab is also employed in the treatment of radiation-induced tissue damage (radionecrosis), where it helps to reduce edema and decrease steroid dependence in affected patients, improving their overall quality of life [146]. Its effectiveness underscores the importance of targeting the TME as a critical aspect of GB treatment [147].

### 6.5. Tumor Treatment Fields (TTFs)

Combined with chemotherapy and radiotherapy, an innovative therapeutic approach that has proven groundbreaking in GB management is TTFs. This innovative therapy involves delivering low-intensity (1–2 V/cm) alternating currents at an intermediate frequency to cancer cells through electrodes placed on the area around the tumor. This technique interferes with the division of actively dividing cells, leading to their apoptosis, while sparing non-dividing cells [148,149].

Research from 2015 showed that the combination of TTFs with TMZ significantly enhances median progression-free survival (PFS) and OS compared to TMZ alone during maintenance therapy, with fewer occurrences of electrical device-related adverse effects [150]. Consequently, current treatment guidelines include TTFs as part of the therapy plan for patients with both newly diagnosed and recurrent GB [151].

Despite these advantages, the use of TTFs faces several challenges. These include the high cost of the device, the necessity for continuous use for at least 18 h a day, and the requirement for hair shaving to ensure proper electrode placement, which can decrease patient compliance [152]. Therefore, while TTFs offer a promising addition to GB treatment, addressing these challenges is essential to maximize their therapeutic benefits.

### 6.6. Supportive Therapy

In view of the fact that patients diagnosed with GB are both undergoing cancer treatment and, due to the tumor localization, experiencing a progressive neurological disease, their care must always involve comprehensive supportive therapy [153]. The primary goal of supportive care is to enhance the patient’s quality of life by addressing the various symptoms and manifestations of the illness [154].

One of the most significant symptoms characterizing GB is seizures, which result from the tumor’s irritation of brain tissue. Effective seizure management typically requires the initiation of antiepileptic medications (AEDs), while their use for prophylactic purposes is generally considered ineffective [155,156]. Within the wide spectrum of AEDs, the most recent drugs, levetiracetam and lacosamide, are often preferred due to their favorable toxicity profile, reduced laboratory monitoring requirements, and minimal drug interactions, making them a suitable option for long-term management [157].

Another frequent and serious complication of GB is vasogenic edema in the peritumoral area, which is often managed with corticosteroids, typically administered alongside gastric protection [158]. There is no established protocol for steroid administration in this context, so their use must be tailored to each patient, with numerous cautions. Long-term corticosteroid use, particularly at doses of ≥20 mg prednisone equivalents daily for up to one month, can in fact cause serious side effects, including osteoporosis and immunosuppression. This increases the susceptibility to infections, including those caused by opportunistic agents such as *Pneumocystis jirovecii* [159]. Therefore, patients undergoing chronic corticosteroid therapy should receive prophylaxis for both osteoporosis, typically with bisphosphonates or other bone-preserving agents (e.g., vitamin D and calcium) [160], and for *Pneumocystis jirovecii* pneumonia, commonly with trimethoprim–sulfamethoxazole [161]. Due to its effectiveness in reducing inflammation and cerebral swelling, which helps alleviate symptoms such as headaches and nausea, dexamethasone represents the steroid of choice in this context. Its main advantages include its potency, long half-life, and high brain penetrance, allowing for low administered doses (e.g., 4–16 mg/day, administered in 1–2 doses) [157,162].

It has also been observed that patients with GB are at an increased risk of experiencing venous thromboembolism (VTE) in the year following surgery, with one of the highest rates among cancer patients. This is due to both the hypercoagulable state induced by the malignancy and the immobility associated with neurological deficits [162]. Managing this risk is extremely complex: studies suggest anticoagulant therapy in patients with brain tumors can increase the risk of intracranial hemorrhage (ICH) [163]. The American Society of Clinical Oncology (ASCO) guidelines, therefore, recommend using anticoagulants only when VTE is confirmed. There is limited research on the optimal anticoagulant for brain tumors; in systemic cancers, low-molecular-weight heparin (LMWH) is generally favored over traditional oral anticoagulants, such as warfarin [164]. Direct oral anticoagulants (DOACs), which include factor Xa and thrombin inhibitors, have also been found to be safe for use in patients diagnosed with brain tumors [165].

Mood disorders, such as depression and anxiety, are prevalent among GB patients due to both the psychological impact of the diagnosis and the biological effects of the tumor and its aggressive treatment [143]. Addressing these psychological challenges is essential, with selective serotonin reuptake inhibitors (SSRIs) and cognitive–behavioral therapy (CBT) being common approaches to effectively manage these conditions [166].

Cognitive impairment is another debilitating consequence of GB, necessitating cognitive rehabilitation programs that involve occupational therapy and memory exercises [167]. In some cases, medications typically used for Alzheimer’s disease, like donepezil or psychostimulants (methylphenidate, modafinil), may also be considered to help improve cognitive function [168,169,170].

Finally, one of the most crucial aspects of supportive care for cancer patients, including those with GB, is pain management. Pain can stem from both the tumor and its various treatments, which are often invasive [171]. A multimodal approach to pain management is typically employed, with opioids playing a central role in achieving effective pain relief. For patients who are new to opioids and report mild to moderate pain, weak opioids such as codeine, hydrocodone, and tramadol are usually recommended as a starting point. There is no significant difference in the efficacy among these drugs [172,173,174]. Weak opioids are often combined with non-opioid analgesic drugs like acetaminophen (paracetamol) or non-steroidal anti-inflammatory drugs (NSAIDs) to enhance pain relief.

For moderate to severe pain, strong opioids such as morphine, oxycodone, and hydromorphone are recommended. It is essential to start with a low dose and gradually adjust it to achieve a balance between effective pain relief and manageable side effects [171].

Cannabis-based medicines (CBMs) have gained increasing attention in recent years, especially following the recent legalization of cannabinoids in many countries. This has spurred interest in their potential role in pain management [175]. In addition to opioids and CBMs, adjuvant therapies can be useful in managing neuropathic pain. These include tricyclic antidepressants (TCAs) like amitriptyline and nortriptyline, serotonin–norepinephrine reuptake inhibitors (SNRIs) such as duloxetine and venlafaxine, and anticonvulsants, primarily gabapentin and pregabalin [176]. These therapies can help alleviate neuropathic pain and further improve the patient’s overall comfort.

## 7. Next-Generation Therapeutics in GB: From Biotechnologies to Smart Delivery Systems

**Figure 4:**
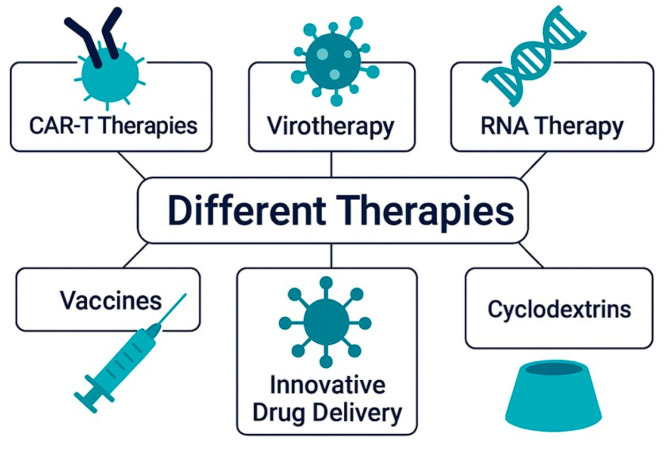
Schematic illustration of different therapies in oncology.

Despite notable advances in oncology, GB remains one of the most lethal and treatment-resistant malignancies. Standard therapies offer limited survival benefits, largely due to the tumor’s invasive nature, molecular heterogeneity, and resistance mechanisms. These limitations have catalyzed the development of innovative, multimodal strategies that combine molecular targeting, immunotherapy, and advanced delivery systems [177,178], shown in Figure 4.

RNA-based therapies are among the most promising innovations in GB treatment, offering highly specific modulation of gene expression [179].

Parallel to RNA approaches, other advanced strategies are gaining traction. Chimeric Antigen Receptor T-cell (CAR-T) therapies [180] reprogram the immune system to target GB-specific markers, and oncolytic viruses (OVs) [181] selectively infect tumor cells while exposing neoantigens to the immune system. Smart drug delivery systems (DDSs), such as nanoparticles (NPs), CYDs, and stimuli-responsive carriers, enable precise delivery across the BBB and ensure controlled drug release within the TME. The true strength of these approaches lies in their synergistic potential, where each component enhances the others, collectively overcoming the limitations of conventional treatments [182,183,184].

Among the various delivery strategies, CYD-based formulations stand out as particularly promising, offering enhanced drug stability, improved brain penetration, and the potential to significantly boost the efficacy of combination therapies. Their unique ability to modulate pharmacokinetics and overcome biological barriers makes them an ideal platform for next-generation GB treatments [22].

This section explores how integrated molecular and nanotechnological strategies are reshaping the therapeutic landscape of GB, paving the way for personalized and more effective treatments, focusing on CYD-based systems.

### 7.1. CAR-T Therapy

Given the limitations of standard therapies, CAR-T cell therapy has emerged as a promising approach for GB [180]. This strategy involves engineering a patient’s T lymphocytes to express chimeric antigen receptors (CARs) that recognize tumor-specific antigens, enabling selective targeting and elimination of malignant cells upon reinfusion [185]. For GB, the most extensively investigated key targets include HER2, Interleukin 13Rα2 Receptor (IL-13Ra2), and Epidermal Growth Factor Receptor Variant III (EGFRvIII), among others [185,186,187,188].

Preclinical studies have demonstrated that HER2-directed CAR-T cells can induce tumor regression and robust antitumor responses in GB and other brain tumor models [189]. However, early clinical trials were limited by systemic toxicities. A second-generation HER2-CAR incorporating the CD28 co-stimulatory domain was evaluated in a phase I trial involving 17 HER2-positive GB patients. Administered without lymphodepletion, the therapy was well tolerated, with no dose-limiting toxicities. HER2-CAR-T cells persisted for up to 12 months in some patients and were associated with partial responses or disease stabilization, with a median overall survival of 11.1 month [190].

IL-13Rα2 is a monomeric receptor aberrantly overexpressed in more than 75% of GB.

Due to its restricted normal tissue distribution and tumor specificity, IL-13Rα2 is considered a highly selective target for CAR-T therapy [191]. L-13Rα2-specific CAR-T cells showed strong cytotoxicity against glioma stem-like and bulk tumor cells [192]. First-generation constructs had limited persistence and required high doses [193]. A second-generation CAR with 4-1BB co-stimulation (IL-13BBζ) improved antitumor activity and persistence [194,195].

EGFRvIII, a tumor-specific variant of EGFR found in approximately 50% of GB cases and absent in normal tissues, offers high specificity and reduced off-target effects compared to targets such as HER2 [196,197,198,199]. EGFRvIII-directed CAR-T cells have shown efficacy in preclinical models, inducing tumor regression and extending survival [200].

Despite promising results in hematologic malignancies [201], CAR-T therapy in GB remains challenging due to antigen heterogeneity, limited trafficking across the blood–brain barrier (BBB), and rapid immunoediting. GB tumors can evade immune surveillance by altering antigen expression, contributing to immunosuppression and resistance [202]. To address these barriers, multi-target CAR-T strategies and combination therapies with immune-enhancing agents are being explored. Enhancements such as the incorporation of microRNA clusters (e.g., miR-17–92) improve CAR-T cell persistence and efficacy, while humanization of the scFv domain reduces immunogenicity and supports long-term T-cell survival [203,204].

### 7.2. Virotherapy

Oncolytic viral therapy (or virotherapy) is an innovative form of immunotherapy that employs genetically engineered OVs to target and destroy cancer cells while also stimulating the body’s immune system to fight against the tumor [181]. These viruses are designed to selectively infect and replicate within tumor cells, sparing healthy cells from damage. The therapeutic impact extends beyond direct tumor lysis: viral activity remodels the immunosuppressive TME, transforming “cold” tumors (immune-excluded) into “hot” tumors (immune-responsive) by triggering immunogenic cell death [205]. This process releases tumor-associated antigens (TAAs), damage-associated molecular patterns (DAMPs), and viral pathogen-associated molecular patterns (PAMPs), which recruit antigen-presenting cells (APCs) to lymph nodes. These APCs activate cytotoxic CD8+ T lymphocytes, which migrate to the tumor site to execute targeted killing [206,207,208].

Currently, two main types of oncolytic viral therapies are under investigation: Replication-Competent Oncolytic Virus Therapy, where viruses selectively infect and replicate within tumor cells, leading to cell lysis and spread to neighboring cancer cells; and Replication-Deficient Oncolytic Virus Therapy, which uses viruses as vectors to deliver therapeutic genes that induce tumor cell death or stimulate antitumor immune responses [185,209].

Clinical trials have investigated more than 20 OVs for GB, including HSV-1 (e.g., G47Δ) [210,211], adenovirus (e.g., DNX-2401) [212], and the polio–rhinovirus chimera PVSRIPO [213].

Despite recent advances, key challenges remain in improving viral targeting, avoiding immune neutralization, and combining OVs with therapies like checkpoint inhibitors or CAR-T cells to establish virotherapy as a next-generation GB treatment [209].

### 7.3. RNA Therapy

An increasingly powerful and versatile strategy in the field of innovative cancer therapies, including GB, is represented by RNA-based therapeutics. These approaches leverage RNA’s central role in gene expression regulation and immune modulation, offering highly specific solutions tailored to the molecular profile of individual tumors. Among the diverse RNA-based modalities, two have emerged as particularly transformative: RNA interference (RNAi) and messenger RNA (mRNA) therapeutics [179].

RNAi enables the selective silencing of genes critical to GB pathogenesis by using small molecules, such as siRNAs and shRNAs. These molecules are engineered to bind with high specificity to overexpressed tumor mRNAs, thereby preventing their translation into functional proteins [214]. This strategy has shown significant promise in targeting key oncogenic drivers in GB, including hyperactivated signaling pathways like EGFR/PTEN/AKT/mTOR. These pathways not only sustain malignant proliferation and invasiveness but also contribute to the notorious resistance of GB to conventional therapies [215]. Preclinical investigations have demonstrated that siRNAs directed against EGFR or anti-apoptotic genes such as Bcl-2 can significantly suppress tumor progression and even restore sensitivity to chemotherapy agents such as taxol [216,217].

mRNA therapeutics offer a novel approach to GB treatment by leveraging host cells to transiently produce functional proteins from synthetic instructions [218,219].

This strategy can stimulate strong antitumor immune responses, as seen with mRNA vaccines encoding tumor-specific antigens like EGFRvIII or patient-specific neoantigens [185,220,221].

Beyond vaccines, mRNA can be engineered to express immune checkpoint inhibitors (e.g., anti-PD-1, anti-CTLA-4), enhancing antitumor immunity [177]. A key innovation is self-amplifying mRNA (saRNA), which includes viral replicase sequences to prolong protein expression, reducing dosing frequency and improving efficacy [222].

Nevertheless, despite these advancements, the clinical translation of RNA therapeutics for GB is hampered by several obstacles. Chief among these is the BBB, which severely restricts the passage of macromolecules into the CNS. Moreover, RNA molecules are unstable and vulnerable to rapid enzymatic degradation in physiological environments [223,224].

### 7.4. Vaccines

Cancer vaccines aim to trigger targeted immune responses against tumor cells by presenting TAAs that activate cytotoxic T lymphocytes. Once administered, these antigens stimulate the adaptive immune system, particularly cytotoxic T lymphocytes, to identify and destroy tumor cells [225,226,227,228].

Vaccine development for GB is hindered by its molecular heterogeneity and immune-evasive microenvironment [229,230]. Genomic alterations complicate the identification of universal targets, leading most vaccine strategies to focus on shared antigens like EGFRvIII, present in ~30% of GB cases and absent in normal tissue [185,220,231].

Rindopepimut, a peptide vaccine targeting EGFRvIII, showed promising results in early-phase trials (ACTIVATE, ACT II, ACT III) with a median OS of 20–22 months. However, the phase III ACT-IV trial failed to show a survival benefit, and EGFRvIII expression was lost in over 50% of recurrent tumors. A subsequent phase II study (ReACT) combining rindopepimut with bevacizumab showed improvements in secondary endpoints but not in progression-free survival [232].

These findings highlight both the potential and limitations of EGFRvIII-targeted vaccines [233].

### 7.5. Innovative Drug Delivery System

DDSs are advanced formulations designed to control the release and distribution of therapeutic agents, overcoming the limitations of conventional immediate-release drugs [234,235]. Unlike conventional forms, which deliver the entire dose rapidly and in an uncontrolled manner—primarily dependent on the physicochemical properties of the active pharmaceutical ingredient, such as solubility, permeability, and stability under physiological conditions—DDSs use technologies such as polymeric matrices, lipid carriers, and osmotic systems to modulate drug kinetics, improve targeting, and reduce side effects [236]. These systems help maintain stable plasma concentrations, enhance patient compliance, and minimize toxicity, especially for drugs with narrow therapeutic windows or short half-lives [235,236].

One of the earliest and most iconic examples of controlled-release technology is the Spansule^®^ capsule, introduced in 1952 by Smith, Kline & French Laboratories. This system utilized drug-loaded granules coated with materials of varying dissolution rates, enabling staggered release over time. By incorporating these granules within a single capsule, Spansule^®^ achieved prolonged therapeutic action and reduced dosing frequency—laying the foundation for modern sustained-release formulations. These include Dexedrine^®^ (dextroamphetamine sulfate) and Contac^®^ 600 (a combination of phenylpropanolamine hydrochloride and chlorpheniramine maleate) [237,238]. Other landmark developments in the evolution of modified release systems include Ocusert^®^, a sustained ocular drug delivery device that relies on diffusion-controlled membranes for the release of pilocarpine in glaucoma treatment, and OROS^®^ technology, which utilizes osmotic pressure to ensure consistent, controlled oral delivery of various therapeutics [239].

These foundational innovations laid the groundwork for a wide and diverse range of modern DDS, which now vary extensively in terms of design, materials, and application, but share the common goal of optimizing drug performance [240,241]. Among these, liposomes, discovered by Alec Bangham in the 1960s, were the first nanosystems to be used in chemotherapy. These phospholipid-based vesicles, resembling cellular membranes, can encapsulate both hydrophilic drugs within their aqueous core and hydrophobic agents within their lipid bilayer, enabling targeted and sustained delivery [242,243,244]. Their biocompatibility and biodegradability minimize systemic toxicity, while surface modifications—such as PEGylation, creating “stealth liposomes”—extend circulation time by evading immune clearance [245]. Clinical applications highlight their transformative potential: AmBisome^®^ reduces nephrotoxicity of amphotericin B in antifungal therapy [246], while Doxil^®^/Caelyx^®^ leverages PEGylated liposomes to enhance tumor accumulation of doxorubicin via the enhanced permeability and retention (EPR) effect, markedly lowering cardiotoxicity [247]. Liposomes also facilitate active targeting through antibody conjugation (immunoliposomes) or ligand attachment (e.g., folate for cancer cells), improving specificity [248]. Beyond oncology, they serve in vaccine development (e.g., virosomes for influenza) and gene therapy by complexing with nucleic acids [249]. Their adaptability across administration routes—intravenous, topical, oral, or inhalational—underscores their role as a cornerstone in modern drug delivery [244].

**Figure 5:**
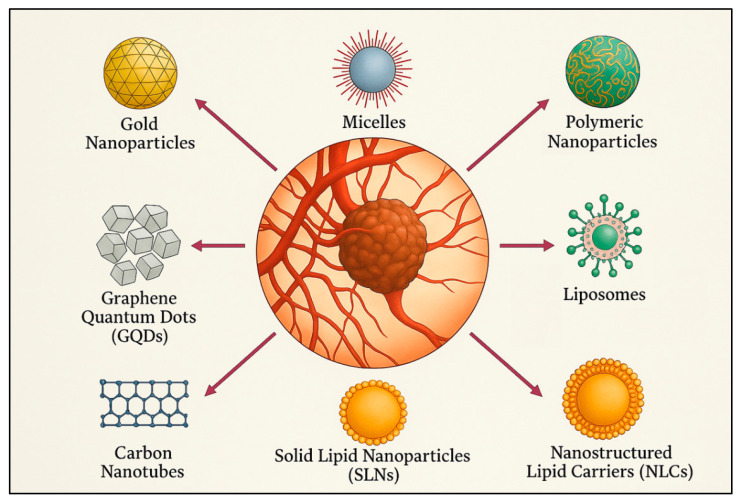
Illustration of various nanosystems employed in enhancing cancer management.

Beyond liposomes, various other nanocarrier systems have been developed and are currently being explored for their potential in cancer therapy (Figure 5).

For instance, inorganic NPs, such as those based on gold or silica, present unique benefits, including the possibility of simultaneous imaging and therapy, thus enabling a theranostic approach [250]. Meanwhile, polymeric micelles formed from amphiphilic block copolymers have proven particularly effective for encapsulating and solubilizing hydrophobic drugs that would otherwise be challenging to deliver via the bloodstream [251].

But in recent years, those that have gained more prominence are polymeric NPs. In these systems, the drug material can be entrapped, encapsulated, or dissolved within a polymeric matrix (nanospheres, NSs), or adsorbed onto the surface of the matrix (nanocapsules, NCs) [252]. They are typically fabricated from biodegradable polymers such as PLGA [253], dextran [254], or chitosan [255], all of which are susceptible to enzymatic degradation. They are particularly effective at protecting labile drugs from premature degradation and enabling both sustained and stimulus-responsive release profiles [256]. Analogously to liposomal systems, their surfaces can be engineered with PEG chains to create “stealth NPs” that evade immune recognition and prolong systemic circulation [257]. Moreover, functionalization with targeting ligands—such as antibodies, peptides, or folate—enables selective delivery to diseased tissues, enhancing therapeutic efficacy while minimizing off-target effects [258]. A notable example includes hyaluronic acid-coated NPs, which have demonstrated particular promise in targeting CD44-overexpressing cancer cells [259,260,261], a feature common in aggressive tumors, including GB [262].

Depot formulations, such as Zoladex^®^ (goserelin acetate), use biodegradable polymeric matrices to release drugs gradually over weeks or months, reducing dosing frequency and maintaining long-term therapeutic effects [236,263]. Their small particle size (<1 µm) also enhances oral bioavailability by facilitating uptake through intestinal mucosa or lymphoid structures like Peyer’s patches [264].

In ocular applications, polymeric NPs can exhibit mucoadhesive properties, depending on the type of polymer used, with chitosan being among the most commonly employed [265,266]. This characteristic allows them to adhere to the eye’s mucous membranes for extended periods, thereby increasing their residence time on the corneal surface. As a result, the drug remains in sustained contact with the target tissue, ultimately enhancing therapeutic outcomes in ophthalmic treatments [265].

Together, these characteristics make polymeric NPs highly versatile tools in modern medicine, capable of improving drug delivery in ways that are both targeted and efficient.

## 8. Cyclodextrins (CYDs)

### 8.1. Discovery: 1891–1911

**Figure 6:**
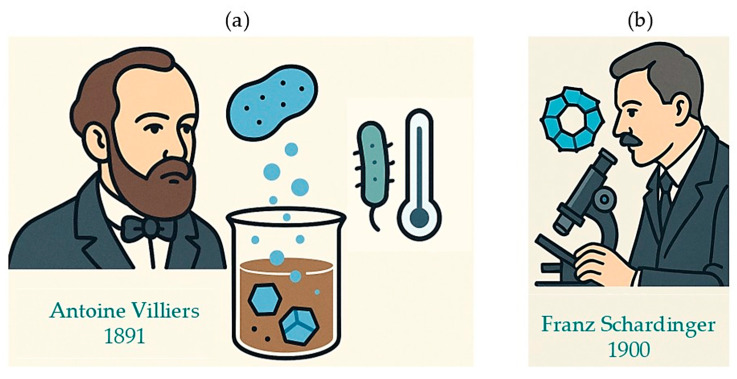
Cartoon-style representation of (**a**) the discovery of CYDs by Antoine Villiers; (**b**) the founding father of CYDs, Franz Schardinger.

The discovery of CYDs dates back to 1891, when Antoine Villiers, a French scientist and pharmacist, observed the formation of unusual crystalline products during his experiments on the enzymatic degradation of carbohydrates. In his study, Villiers incubated 50 g of potato starch in 1 L of water at 100 °C, inoculated with Bacillus amylobacter, and maintained the mixture at 40 °C for several days (Figure 6a). At the end of the incubation, he noted the presence of crystalline substances, which he presented to the French *Académie des Sciences* in February 1891 [267]. In a subsequent communication in June of the same year, Villiers described these products as highly crystalline structures, possibly intermediate decomposition products of starch. After refining the experimental conditions and purifying the samples in hot water, he obtained small, bright crystals, which were later identified as α-CYD and β-CYD. Villiers recognized that these dextrins exhibited properties distinct from known saccharides and polysaccharides. Due to their structural resemblance to cellulose, he proposed the term “cellulosines” to describe them [267].

The formal recognition and characterization of CYDs, however, are attributed to Franz Schardinger, an Austrian chemist and bacteriologist, who is widely regarded as the founding father of CYDs (Figure 6b). In the early 1900s, Schardinger studied the formation of these cyclic dextrins—initially referred to as “krystallisiertes Dextrin”—as degradation products of starch under enzymatic and thermal conditions [268,269].

In honor of his contributions, CYDs were commonly referred to as “Schardinger dextrins” until the 1970s.

The discovery of CYDs marked a turning point in carbohydrate chemistry. From Villiers’ first crystalline products to Schardinger’s structural insights, these early breakthroughs transformed starch derivatives into a new molecular class, laying the foundation for a century of innovation.

### 8.2. CYD Chemistry

**Figure 7:**
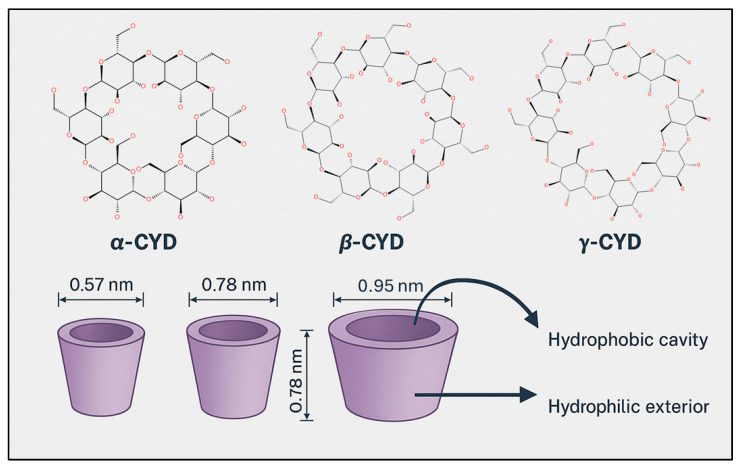
Functional structural scheme and geometric dimensions of α-CYD, β-CYD, and γ-CYD.

Cyclodextrins (CDs) are cyclic oligosaccharides composed of α-1,4-linked glucopyranose units, enzymatically synthesized from starch via the action of CYD glucanotransferase (CGTase). This extracellular enzyme, predominantly produced by Bacillus species, catalyzes an intramolecular transglycosylation reaction that cleaves and rearranges starch chains to form cyclic structures. Depending on the number of glucose units, this process yields α-CD (6 units), β-CD (7 units), and γ-CD (8 units), each with distinct cavity sizes and physicochemical properties (Figure 7) [270]. The enzymatic production is highly efficient and selective, and industrial processes have optimized it further by employing thermostable CGTases, which improve reaction stability and product yield under varying conditions. Structurally, CDs exhibit a characteristic truncated cone shape, with a hydrophilic outer surface and a hydrophobic inner cavity. This unique geometry enables them to form inclusion complexes with a wide range of hydrophobic guest molecules, making them valuable carriers in pharmaceutical, food, and cosmetic applications [271].

These are the most commonly studied and commercially available forms due to their relative ease of production and purification. The existence of larger ring structures containing nine to twelve glucose units—referred to as δ-, ζ-, ξ-, and η-CYDs—was first documented in 1961, although these forms are rarely used due to their low yield, poor stability, and difficulty in isolation [272]. Conversely, CYDs with fewer than six glucose units do not form naturally, as the steric strain and unfavorable ring tension prevent successful cyclization and stable ring closure [273].

These molecules have two key features: an exterior hydrophilic surface covered in many hydroxyl groups and a hydrophobic internal cavity whose size is determined by the amount of glucose units [274].

By virtue of such characteristics, CYDs can provide favorable microenvironments for forming inclusion complexes with organic molecules and drugs in different stoichiometric ratios (1:1; 2:1; 1:2; 2:2, Figure 8) [275,276,277,278,279,280]. This encapsulation ability allows CYDs to modify the physicochemical properties of the guest molecules [281,282,283,284]. Host–guest recognition is a selective process, relying on a size/shape match in which host molecules are “trapped” only partially or entirely in the internal lipophilic cavity of CYDs [285,286,287]. The formation of these complexes involves weak interactions, such as hydrogen bonds, van der Waals forces, and electrostatic forces, which stabilize the complex while allowing for the release of the molecules [288,289]. Complexation is moved by thermodynamic gains produced by the formation of hydrophobic and van der Waals interactions and exclusion from the cavity of high-energy water molecules [290]. In solution, some water molecules are entrapped within the CYD cavity, forming energetically unfavorable interactions with the internal apolar cavity. At the same time, water molecules arranged around the apolar drug act as “iceberg” structures, restricting their mobility and entropy. When the apolar guest molecule penetrates into the CYD cavity, forming physical interactions, the constrained water molecules are released into the bulk solution, increasing entropy and producing a net thermodynamic gain. This entropy-driven process and physical host–guest interactions promote the spontaneous formation of the inclusion complex. However, a volumetric compatibility between the drug and the cavity is needed to obtain stable and complete inclusion, with high complexation efficiency. This latter is a key parameter in therapeutic applications of CYD inclusion complexes, because it determines the amount of excipient (CYD) required to administer a specific drug dose. Poor host–guest compatibility requires large amounts of CYD to improve solubility and stability of the drug, enhancing costs and potential toxicity for parenteral or high-dose oral administrations [291].

In addition to their use in pharmaceuticals, CYDs are essential in analytical chemistry and food science. To increase their stability and cover up unwanted aromas or odors, CYDs are used in the food sector to encapsulate flavors, perfumes, and food additives [292,293]. Beyond this, CYDs are efficient adsorbents that remove toxins and contaminants from food and drink, promoting food safety and quality assurance [294].

CYDs are used in analytical chemistry to separate and purify organic substances, especially enantiomers. CYD-based stationary phases allow for high selectivity in resolving racemic mixtures, simplifying product analysis [295,296]. Furthermore, CYDs function as flexible chiral selectors in chromatography and capillary electrophoresis, offering accurate and exact analysis of complicated combinations [297,298].

Natural CYDs exhibit a hydrophilic surface, but tend to form aggregates, especially β-CYD, through intramolecular hydrogen bonds, resulting in low solubility in an aqueous environment, which limits their use [299]. This low solubility can result in nephrotoxicity, as insoluble CYD or CYD–cholesterol complexes may accumulate as crystals in the kidneys [300]. To overcome these limitations and enhance the action of CYDs, the primary strategy has been to modify their chemical structure [301,302].

CYDs possess three different hydroxyl groups that can be chemically modified: the 6-OH group on the upper edge, which is the most accessible for modification; the more acidic 2-OH groups; and the less accessible 3-OH groups on the lower edge. Due to the varying reactivity of these hydroxyl groups, it is possible to selectively substitute them, leading to the synthesis of over 11,000 CYD derivatives. These derivatives can incorporate a wide range of functional groups (e.g., amino acids, peptides, amines, and aromatic compounds) to improve water solubility and enhance their ability to form inclusion complexes. Additionally, the structural complexity of CYDs can be further expanded by creating inclusion complexes between branched CYDs and guest molecules, facilitating the development of advanced supramolecular systems such as polymers, metal–organic frameworks, and hydrogels [303,304,305].

Despite its low solubility in water, β-CYD has gained increasing pharmaceutical relevance due to the loss of its crystalline solid state, achieved through chemical modifications that significantly enhance its solubility [14,306,307]. It has been hypothesized that random substitution of any hydroxyl group can disrupt the stable hydrogen bonding system around the edges of β-CYD, leading to increased solubility in water. Consequently, several pharmaceutical derivatives of CYDs have been developed, including sulfobutyl ether β-CYD (SBE-β-CYD), hydroxypropyl derivatives of β-CYD, randomly methylated β-CYD (RAMEB), and branched CYDs such as maltosyl-β-CYD [308,309].

These modifications have not only expanded the utility of CYDs in pharmaceutical applications but also opened new avenues for their use in other fields, such as the food industry, analytical chemistry, agriculture, and environmental protection, demonstrating the vast potential of these versatile compounds [310].

CYDs combine a hydrophilic exterior with a hydrophobic cavity, enabling selective molecular encapsulation. This unique architecture underpins their role in solubility enhancement and complex formation, making them indispensable in pharmaceuticals, food science, and analytical chemistry. From simple starch rings to powerful supramolecular tools, CYDs embody a cornerstone of modern formulation science.

### 8.3. Cyclodextrin Derivatives: HP-β-CYD and SBE-β-CYD

**Figure 9:**
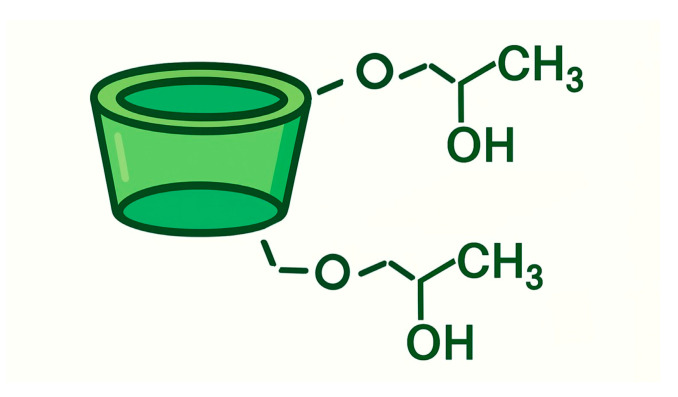
Cartoon-style illustration of HP-β-CYD showing its structural features and side-chain modification.

HP-β-CYD (Figure 9) is a neutral, non-ionic derivative of β-CYD, obtained by the hydroxyalkylation of hydroxyl groups at the 2-, 3-, and/or 6-positions of the glucose units.

The introduction of hydroxypropyl groups disrupts the crystalline structure of native β-CD, resulting in a highly water-soluble compound (solubility > 100 mg/mL) and improved amorphous characteristics [311,312,313]. Its amphiphilic nature arises from the hydrophobic cavity, which can host non-polar molecules, and the hydrophilic exterior, which ensures compatibility with aqueous environments [314]. This duality enables HP-β-CYD to form stable inclusion complexes with poorly soluble drugs, enhancing their solubility, dissolution rate, and bioavailability, while maintaining their chemical integrity [315,316].

Being neutral, HP-β-CYD does not carry ionic charges, which contributes to its low toxicity and excellent safety profile, making it suitable for various routes of administration, including oral, parenteral, ophthalmic, rectal, and topical formulations [317].

Gaspar et al. [318] demonstrated that HP-β-CYD significantly reduces age-related accumulation of lipofuscin in neuronal tissues through a cholesterol-associated mechanism. In their study, aged mice treated systemically with HP-β-CYD showed a marked decrease in lipofuscin deposits, particularly in the hippocampus and cortex—regions commonly affected in neurodegenerative diseases. The authors attributed this effect to HP-β-CYD’s ability to mobilize intracellular cholesterol, thereby enhancing lysosomal function and promoting the clearance of undegraded cellular waste. Furthermore, HP-β-CYD-treated mice exhibited improved cognitive performance in behavioral tests, suggesting a potential neuroprotective role. These findings support the therapeutic promise of HP-β-CYD in combating age-related neurodegenerative conditions linked to lipid metabolism dysfunction.

Loftsson et al. [319] demonstrated that HP-β-CYD, a widely used solubilizing excipient, exhibits favorable renal clearance properties that may contribute to renal protection. Following parenteral administration, HP-β-CYD is rapidly and almost entirely excreted unchanged via glomerular filtration, with approximately 90% eliminated within 6 h and 99% within 12 h in individuals with normal kidney function. Notably, HP-β-CYD has a small volume of distribution (≈0.2 L/kg) and a short half-life (≈1.7 h), indicating minimal tissue accumulation and efficient renal elimination. In pharmacokinetic studies, co-administration of HP-β-CYD with dexamethasone led to increased renal clearance of the drug, likely due to the formation of hydrophilic HP-β-CYD–drug complexes that reduce tubular reabsorption. These findings suggest that HP-β-CYD not only facilitates drug solubilization and delivery but may also enhance renal drug elimination, potentially reducing nephrotoxic risk by limiting drug accumulation in renal tissues.

A phase 2a open-label clinical trial [320] has been conducted to evaluate the safety and efficacy of a single intravenous dose of HP-β-CYD in patients with type 2 diabetic kidney disease (DKD) and proteinuria. Sponsored by ZyVersa Therapeutics, the study enrolled eight participants across U.S. sites and focused on changes in urinary albumin-to-creatinine ratio over 12 weeks, as well as treatment-emergent adverse events. HP-β-CYD functions as a cholesterol efflux mediator, promoting lipid clearance from renal cells via ABCA1 and ABCG1 transporters. Audiometric assessments were included due to potential otologic effects. Although no peer-reviewed publications are currently available, the study’s outcomes may provide promising evidence for the role of lipid modulation in slowing DKD progression and support further development of HP-β-CYD as a novel therapeutic strategy.

**Figure 10:**
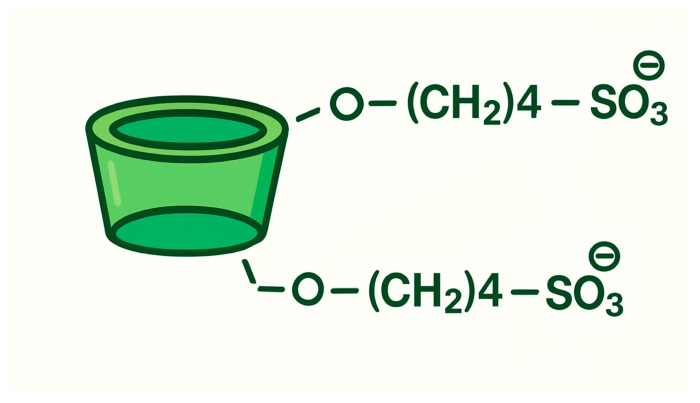
Cartoon-style illustration of SBE-β-CYD showing its structural features and side-chain modification.

SBE-β-CYD (Figure 10) is an anionic CYD derivative of β-CYD with high water solubility.

The sulfobutylation process can occur at the primary hydroxyl group (C6) and the secondary hydroxyl groups (C2 and C3) of the glucose units. Each of these positions exhibits distinct chemical reactivity:

- The primary hydroxyl group at C6 is the most nucleophilic, making it highly reactive under strongly alkaline conditions.
- The secondary hydroxyl group at C2 is the most acidic, and under mildly basic aqueous conditions, it tends to react more readily than C6.
- The secondary hydroxyl group at C3, however, is sterically hindered, making it the least accessible and most difficult to modify.

As a result, the degree of substitution (DS), the average number of sulfobutylether groups per CYD molecule varies depending on the reaction conditions. Typically, modifications at C3 occur only after substantial substitution at C6 and C2, due to its lower reactivity and steric constraints [321].

This selective substitution pattern is crucial for tuning the physicochemical properties of SBE-β-CD, including its complexation capacity, solubility, and biocompatibility. The presence of negatively charged sulfonate groups also contributes to reduced aggregation, lower membrane interaction, and minimized hemolytic and nephrotoxic risks, making SBE-β-CD particularly suitable for intravenous drug delivery systems [322].

SBE-β-CYD has demonstrated organ-protective properties when used to form inclusion complexes with drugs known to cause significant side effects. These protective effects are particularly evident in formulations designed to mitigate renal, cardiac, and cellular toxicity.

In 2016, Rowe et al. [323] showed that the addition of small amounts of SBE-β-CYD to iohexol, a contrast agent, in a 40:1 ratio (iohexol:SBE-β-CYD) significantly protected rodent kidneys from contrast-induced acute kidney injury. The proposed mechanism involves protein binding, where SBE-β-CYD interacts with hydrophobic amino acid side chains of key proteins. This interaction disrupts the proteins’ ability to bind to mitochondrial membranes or aggregate, thereby preventing pore formation in the mitochondrial membrane and blocking apoptosis.

Further research by the same team in 2021 [324] revealed that the SBE-β-CYD-iohexol complex was more effective than iohexol alone in preserving cardiomyocyte integrity and maintaining myocardial function during ischemic events. These findings suggest a broader protective role of SBE-β-CYD beyond renal applications. The formulation is currently undergoing Phase II clinical trials, indicating its potential for therapeutic use.

In a separate study, Fliszar-Nyul et al. [325] demonstrated that SBE-β-CYD could mitigate the cytotoxic effects of chlorpromazine, an antipsychotic drug. In dose-dependent cellular assays, SBE-β-CYD reduced chlorpromazine-induced loss of cell viability, and in animal models, it lowered mortality rates. The protective effect is attributed to the formation.

Chemical modifications have elevated CYDs from simple excipients to advanced functional carriers. HP-β-CYD combines high solubility with exceptional safety, enabling efficient drug solubilization and even showing neuroprotective potential, while SBE-β-CYD introduces ionic features for improved compatibility and organ protection. These derivatives exemplify the evolution toward multifunctional drug delivery systems.

### 8.4. CYD and Inclusion Complex Toxicity

CYDs are Generally Recognized as Safe (GRAS) ingredients. However, toxicity can occur at high doses or following inappropriate routes of administration for a specific CYD derivative, including cytotoxicity, ototoxicity, and membrane-disrupting effects [326]. In general, oral administration of low doses of native β-CYD is non-toxic [327], whereas high doses are associated with adverse effects, such as kidney damage, nausea, vomiting, and diarrhea [291]. Similar adverse effects have been reported for other native CYDs. LD_50_ of β-CYD determined after acute oral administration in rats and mice is <12,500 mg/kg, where the parent γ-CYD shows LD_50_ higher than 18,000 mg/kg. Parenteral administration of native β-CYD is prevented by its hemolytic activity related to its ability to extract cholesterol from lipid rafts of red blood cell (RBC) membrane, producing their deformation [328]. It is also associated with nephrotoxicity, due to its low water solubility that can lead to its tubular crystallization in the kidney. Its LD_50_ for i.v. administration is 788 mg/kg, while native γ-CYD exhibits a safer profile, with LD_50_ > 3750 mg/kg. Chemical modification of β-CYD in more soluble carriers, such as SBE-β-CYD and HP-β-CYD, offers an improved safety profile, non-inducing nephrotoxicity and hemolytic effects [329], thus offering a valid carrier in oral and parenteral administration. Nevertheless, solubility must not be the only consideration in evaluating CYD toxicity. For example, methylated-β-CYDs are very-water-soluble agents but they are associated with high toxicity due to their ability to extract lipids and cholesterol from viable membranes, leading to cell damage [330]. Severe nasal mucosal damage was observed following administration of 20% randomly methylated-β-CYD in rats, while 10% was non-toxic.

The formation of inclusion complexes can alter the safety profile of the included drug, improving or reducing its toxicity [331], because of the change in solubility and permeation. Improved solubility can lead to high concentration peaks (Cmax), improving the toxicity, but the slow release of the drug observed in some cases leads to lower Cmax, reducing acute systemic toxicity. The inclusion of an irritating drug can reduce its action on mucosa or skin [332]. Furthermore, complexation can mask unpleasant tastes or smells of drugs or nutraceutical products [333].

Although generally safe, CYDs show hemolytic and nephrotoxic risks, while chemical modifications like HP-β-CYD and SBE-β-CYD greatly improve safety. Structural modifications significantly reduce these risks, but safety depends on more than solubility—methylated derivatives remain highly damaging to membranes. Inclusion complexes can modulate drug toxicity, either reducing irritation or altering systemic exposure, making safety evaluation a multidimensional challenge.

### 8.5. CYDs Regulatory Status

The European Medicines Agency (EMA) has approved native CYDs as excipients and are “Generally Regarded As Safe” by the U.S. Food and Drug Administration (FDA) [317].

Among native CYDs, α-CYD is approved only for parenteral use, β-CYD is approved for oral, rectal, dermal, and ocular use, and γ-CYD is approved for oral and dermal use. Among derived CYDs, SBE-β-CYD is approved in parenteral and oral formulations, whereas HP-β-CYD is one of the safest chemically modified CYDs and is approved for use in parenteral, oral, rectal, dermal, and ocular pharmaceutical formulations. Because methylated CYDs are more likely to produce hemolysis and are more lipophilic, they are less biologically safe. As a result, pharmaceutical companies are prohibited from using the majority of methylated CYDs derivatives. However, one of these molecules is permitted for external use: nasal and ocular preparations contain RAMEB, which is authorized for topical delivery [317].

The nasal formulations produced with CYDs evidence a great degree of adaptability in terms of enhancing the local effects of drugs, or facilitating their systemic or CNS absorption [334].

CYDs enjoy broad regulatory acceptance, with EMA and FDA classifying them as safe excipients. Native forms have route-specific approvals, while derivatives like HP-β-CYD and SBE-β-CYD stand out for their versatility and safety across multiple formulations. In contrast, methylated CYDs face strict limitations due to toxicity risks, with only topical use permitted. This regulatory framework reflects both opportunity and caution.

### 8.6. Mechanisms of Solubility Enhancement by CYDs

CYDs enhance the solubility of poorly water-soluble compounds through a multifaceted mechanism centered on the formation of non-covalent inclusion complexes. Their truncated cone-shaped structure features a hydrophobic inner cavity and a hydrophilic outer surface, allowing CYDs to encapsulate lipophilic moieties of guest molecules while remaining soluble in aqueous environments. Upon complexation, the guest molecule is partially or fully accommodated within the hydrophobic cavity of the CYD, which disrupts its crystalline lattice and reduces the energy barrier for dissolution. This amorphization effect leads to faster wetting, disintegration of the drug particles, and may also mitigate potential toxicological effects by limiting local accumulation or irritation [335].

This process often induces a transition from a crystalline to a more disordered, amorphous-like state. This structural disruption reduces the lattice energy that normally stabilizes the crystalline form, thereby lowering the energy barrier for dissolution. The amorphization effect is thermodynamically favorable, as it increases the system’s entropy and facilitates molecular dispersion in aqueous environments. Molecular dynamics simulations have confirmed that the inclusion of hydrophobic guests like cholesterol into β-CYD, HP-β-CYD, or M-β-CYD cavities leads to the displacement of high-energy water molecules from the hydrophobic core. These confined water molecules are energetically unfavorable due to restricted hydrogen bonding and mobility. Their release upon guest binding results in a net decrease in free energy, making the complexation process spontaneous and entropically driven.

**Figure 11:**
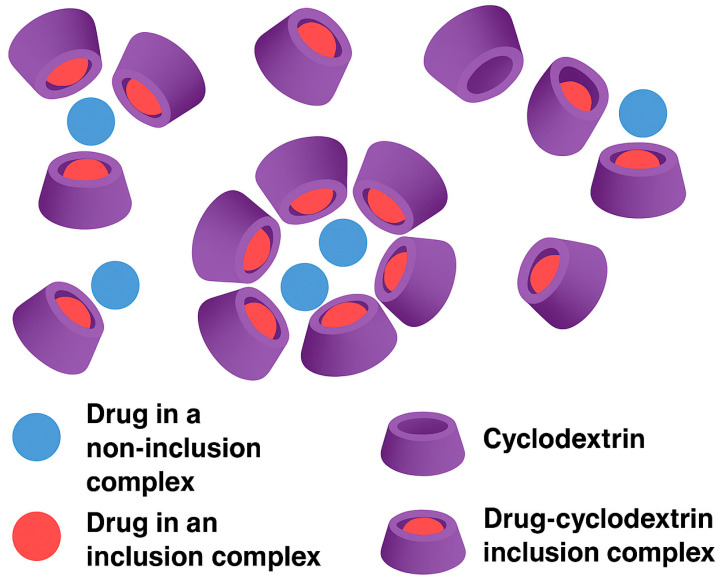
Schematic representation of drug–CYDinteractions showing the formation of inclusion and non-inclusion complexes. Cyclodextrins (purple truncated cones) can encapsulate drug molecules within their hydrophobic cavity (red spheres, inclusion complex) or interact with them externally without cavity penetration (blue spheres, non-inclusion complex).

In addition to inclusion complexes, CYDs can also form non-inclusion complexes [336,337,338] (Figure 11), where the drug interacts with the external surface of the CYD rather than entering its hydrophobic cavity. This phenomenon typically occurs with large or highly polar molecules that cannot fit into the toroidal cavity. Stabilization of these complexes relies on hydrogen bonding, electrostatic interactions, and van der Waals forces between the hydroxyl groups of the CYD and the polar regions of the drug. Although these complexes do not involve true encapsulation, they can significantly influence the physicochemical properties of the active pharmaceutical ingredient, improving solubility and stability without altering its molecular conformation. Understanding these supramolecular interactions is essential for designing advanced drug delivery systems, as non-inclusion complexes offer an alternative strategy for enhancing bioavailability and controlling release profiles in formulations where cavity size or polarity mismatch prevent inclusion complexation.

Additionally, the binding of cholesterol induces conformational changes in the CYD structure, such as cavity expansion and increased sphericity, which further stabilize the inclusion complex and enhance solubilization kinetics. These findings support the notion that CYD-mediated complexation not only improves solubility but also promotes a more dynamic and disordered molecular state that favors dissolution [339,340].

The efficiency of this process depends on several factors, including the type of CYD (α-, β-, γ-, or modified derivatives), the size and polarity of the guest molecule, and the presence of functional groups that favor host–guest interactions such as hydrogen bonding, van der Waals forces, and hydrophobic interactions [341,342]. In some cases, CYDs may also act synergistically with co-solvents or surfactants, enhancing solubility beyond what is achievable by complexation alone.

Overall, CYDs represent a versatile and biocompatible strategy for improving the aqueous solubility, dissolution rate, and stability of challenging drug candidates, making them valuable tools in pharmaceutical formulation and drug delivery.

CYDs enhance solubility through inclusion complexation, disrupting drug crystallinity and creating amorphous states that favor dissolution. This process is entropically driven, as guest binding expels high-energy water molecules from the cavity, lowering free energy. Structural flexibility and non-covalent interactions make CYDs highly adaptable, enabling synergy with co-solvents and surfactants. These mechanisms position CYDs as powerful tools for improving the bioavailability of poorly soluble drugs.

### 8.7. CYDs in GB Treatment

In the last few years, CYDs have attracted considerable interest due to their unique structural characteristics, which are extensively utilized to enhance the performance of active molecules [343]. CYDs are primarily employed to deliver lipophilic drugs, improving their solubility in and stability in an aqueous environment [344]. Additionally, CYDs can cross the BBB, making them promising for the treatment of brain diseases, particularly due to their ability to interact with lipids [345].

CYD-based drug delivery systems have gained increasing attention in oncology due to their ability to enhance solubility, stability, and bioavailability of poorly soluble anticancer agents. Although no clinical trials have yet explored CYD-based formulations specifically for GB, several preclinical studies have demonstrated their potential in this context [346]. CYDs have been successfully incorporated into nanoparticles, liposomes, and conjugates to facilitate targeted delivery and controlled release of chemotherapeutics such as doxorubicin, paclitaxel, and camptothecin [347]. These systems can be further functionalized with ligands or stimuli-responsive components to improve tumor specificity and reduce systemic toxicity [348]. In GB models, nanoscale CD-based carriers have shown promise in overcoming the blood–brain barrier and enhancing drug accumulation within tumor tissue [347]. While these findings are encouraging, translation into clinical applications remains pending, highlighting the need for further investigation and development of CD-based nanocarriers tailored for GB therapy.

The data reported in the literature are summarized below.

#### 8.7.1. Inclusion Complexes

Najlaoui et al. [349] employed RAMEB to form a inclusion complex with N-[4-ferrocenyl,5-5-bis(4-hydroxyphenyl)-pent-4-enyl]-succinimide (SuccFerr), a compound with notable antiproliferative effects demonstrated in vitro. The selection of RAMEB was motivated by the need to enhance the water solubility of this compound, a factor essential for improving the drug’s bioavailability and therapeutic efficacy in biological systems. The complex was prepared in a 1:2 molar ratio of CYD to SuccFerr, resulting in a structure that significantly increases the solubility of the molecule. SuccFerr alone has exhibited remarkable antitumor activity due to the presence of the pharmacophore [ferrocene-ene-phenol] [350], which is known to disrupt cellular redox balance and increase oxidative stress. Specifically, SuccFerr induces apoptosis in cancer cells by elevating reactive oxygen species (ROS) levels, which leads to cellular damage and ultimately cell death [351,352]. Importantly, SuccFerr shows selective cytotoxicity, displaying higher toxicity toward cancer cells than normal cells. Its effects are particularly pronounced in aggressive cancer types, such as GB, where it disrupts mitochondrial function and DNA integrity, resulting in significant reductions in cell viability. Complexation with RAMEB-CYD not only improves the solubility of SuccFerr but also enhances cellular uptake, which may amplify these therapeutic effects. The formation of the complex was confirmed through X-ray diffraction (XRD) analyses and differential scanning calorimetry (DSC), which verified its stability and crystalline structure. These analyses revealed alterations in thermal and crystallographic profiles, indicating effective interaction between RAMEB-CYD and SuccFerr. As reported by Najlaoui et al. [349], the release profile study of the CYD–SuccFerr complex showed a marked increase in drug availability: over 80% of SuccFerr was released into the external phase within 10 h, compared to free SuccFerr, which exhibited negligible release. The anticancer efficacy of the CYD–SuccFerr complex was subsequently tested in vitro on U87 and F98 GB cells, as well as on various other cancer cell lines. The complex demonstrated superior effectiveness against GB cells relative to other types, suggesting potential for targeted therapeutic benefits. Following these promising in vitro results, the complex underwent further in vivo analysis using rat models. Based on preliminary toxicity studies, the optimal therapeutic dose for GB treatment was determined to be 1 mg/kg, balancing safety and efficacy. In vivo investigations confirmed that the CYD–SuccFerr complex significantly inhibited tumor growth and reduced the number of proliferating cells within tumors. Toxicity assessments indicated that the complex is relatively safe, as no lethality or severe adverse effects were observed in rodents. Nevertheless, some changes in blood ion levels and liver enzyme activities were noted, underscoring the need for further research to understand the clinical implications fully. These results highlight both the potent anticancer potential of the CYD–SuccFerr complex and the importance of continued studies to ensure its safety and efficacy in therapeutic applications.

In a recent investigation [353], the anticancer potential of cannabidiol (CBD), a prominent phytocannabinoid, was explored against both GB and rhabdomyosarcoma (RMS), another aggressive and treatment-resistant cancer. Due to CBD’s inherent limitations, such as poor solubility, low bioavailability, and instability, the study aimed to enhance its therapeutic efficacy by forming inclusion complexes with various CYDs. Specifically, HP-β-CYD, RAMEB, 2,6-di-O-methyl-β-CYD (DM-β-CYD), and 2,3,6-tri-O-methyl-β-CYD (TM-β-CYD) were used to improve the physicochemical properties of CBD. Solutions of CBD/RM-β-CD and CBD/HP-β-CD were prepared by dissolving crystalline CBD in aqueous CD solutions (dH_2_O) to achieve final molar ratios of approximately 1:5 and 1:37, respectively. In contrast, crystalline CBD was added to aqueous DM-β-CD or TM-β-CD solutions at a host-to-guest molar ratio of 1:1.

In vitro results on the A172 GB (ECACC 88062428) and TE-671 (ECACC 89071904) RMS cell lines demonstrated that CBD encapsulated in CYD exhibited significantly enhanced cytotoxic effects compared to free CBD or CBD solubilized in conventional solvents like DMSO. Among the complexes tested, CBD/HP-β-CYD showed the highest anticancer activity, followed by CBD/RAMEB-CYD. The cytotoxic effect was dose- and time-dependent, with notable differences observed at various concentrations and exposure times. The IC_50_ values confirmed the superior in vitro activity of CBD/HP-β-CYD and CBD/RAMEB-CYD complexes, highlighting their potential for enhanced therapeutic efficacy. Structural analysis, including X-ray crystallography and molecular dynamics simulations, revealed that the methylated CYDs, DM-β-CYD and TM-β-CYD, exhibited the best inclusion capacity for CBD, improving its solubility in aqueous solutions. These findings emphasize the role of CYDs in enhancing CBD’s bioavailability and stability, potentially overcoming the pharmacokinetic limitations that have hindered its clinical use. The study suggests that CBD encapsulated in CYDs, particularly in complexes with HP-β-CYD, could offer a promising novel strategy for treating highly aggressive cancers like GB and RMS.

Similarly, Qu et al. [354] addressed the limited therapeutic options for GB by investigating disulfiram (DSF), an FDA-approved acetaldehyde dehydrogenase inhibitor traditionally used to manage alcohol dependence but with emerging anticancer applications. A primary limitation of DSF, however, is its low water solubility, which restricts bioavailability and therapeutic efficacy. To overcome this, the researchers developed an inclusion complex of DSF with HP-β-CYD, achieving a remarkable enhancement in DSF’s aqueous solubility—approximately a 2450-fold increase, as confirmed by high-performance liquid chromatography (HPLC) analysis. This increase in solubility is crucial for potential intravenous or intranasal applications, especially for brain-targeted therapies. The study further demonstrated that when this complex was combined with copper, it significantly increased the antitumor activity of DSF, particularly against glioma cells. Cu is integral to DSF’s mechanism of action, as it forms a complex with DSF that acts as a potent proteasome inhibitor, elevating intracellular levels of reactive oxygen species (ROS) and promoting apoptosis specifically in cancer cells. This copper-dependent mechanism causes oxidative stress, selectively targeting cancer cells due to their higher susceptibility to ROS-induced damage [355,356]. Notably, the study explored intranasal administration of the DSF/HP-β-CYD/Cu complex, which provided direct access to the brain, bypassing the BBB and avoiding first-pass hepatic metabolism. This administration route demonstrated superior efficacy in glioma-bearing male rats, significantly inhibiting tumor growth and migration, promoting apoptosis within the tumor, and increasing the median survival time compared to other administration methods. These findings emphasize the potential of intranasal delivery as a minimally invasive and efficient approach for brain-targeted drug delivery [357]. The safety of this system was evaluated through 3-(4,5-dimethylthiazol-2-yl)-2,5-diphenyltetrazolium bromide (MTT) assays on the C6 cell line, confirming cellular viability at effective dosages, while histological analysis of brain tissue revealed no significant damage, supporting the potential clinical application of DSF as a repurposed anticancer treatment. Comprehensive characterization of the DSF-HP-β-CYD complex was conducted using DSC, scanning electron microscopy (SEM), Fourier-transform infrared spectroscopy (FTIR), and XRD analyses, confirming the stable encapsulation of DSF within the CYD matrix. This study highlights DSF’s potential in oncology, especially for cancers resistant to conventional treatments and those requiring targeted BBB penetration.

As previously discussed, TMZ is a cornerstone in the management of GB; however, its therapeutic efficacy is limited by several challenges, including its short half-life, poor penetration of the BBB, and the development of drug resistance. In a study by Krzak et al. [358], CYDs were investigated as potential drug delivery systems aimed at enhancing TMZ’s solubility, stability, and therapeutic efficacy, while mitigating its side effects. The study examined several CYD derivatives, including 6-monodeoxy-6-monoamino-β-CYD hydrochloride (β-CYDamine), β-CYD derivatives with galactosamine and triazole rings (βCYDgal), and standard β-CYD. These derivatives demonstrated the ability to form 1:1 inclusion complexes with TMZ, which significantly improved its solubility and bioavailability. Stability tests conducted at physiological pH (7.4) and slightly acidic pH (5.5) revealed that the TMZ-βCYDgal complex exhibited the highest stability, highlighting its potential as a superior carrier for enhancing TMZ solubility and pH-dependent availability. Furthermore, the study noted that CYDs can interact with cholesterol, a property that may enhance cellular uptake of TMZ, suggesting that CYDs could play a crucial role in overcoming barriers to effective drug delivery.

The exploration of CYD-based formulations extends beyond conventional chemotherapeutic agents like TMZ. As demonstrated in multiple studies, CYDs have proven effective in improving the delivery and therapeutic efficacy of other treatment modalities, such as photodynamic therapy (PDT). PDT has emerged as a promising approach for treating GB due to its targeted mechanism of action, which utilizes light-activated photosensitizers to generate ROS that induce selective tumor cell death [359]. However, the clinical utility of PDT is often hindered by the hydrophobic nature and poor bioavailability of many photosensitizers [360]. To address these challenges, Mihoub et al. [361] investigated the conjugation and encapsulation of photosensitizers with β-CYD to enhance their solubility, stability, and cellular uptake. The study formulated and analyzed three different supramolecular systems for their efficacy in reducing self-aggregation and improving bioavailability. Among the tested systems, the β-CYD-conjugated in a 1:2 molar ratio and the formulation showed superior performance, demonstrating enhanced stabilization and cellular uptake of the photosensitizer. The dark cytotoxicity and phototoxicity of the tested compounds were performed on human GB U87 and U251 cells after incubation in the dark (dark cytotoxicity) or after exposure to light (phototoxicity). This increased uptake was attributed to β-CYD’s unique ability to interact with biological membranes, facilitating more effective transport and internalization. The structural stability of these β-CYD–photosensitizer complexes was confirmed through XRD and molecular dynamics simulations, verifying their integrity and functionality. The results indicated that the β-CYD-based system remained stable and effectively produced ROS upon light activation, significantly improving the therapeutic outcomes of PDT and overcoming the solubility and aggregation limitations typically associated with conventional photosensitizers.

Further supporting the versatility of β-CYD in enhancing drug delivery, Gularte et al. developed an inclusion complex of β-CYD with curcumin (CUR), embedded within a cationic starch and poly(vinyl alcohol) membrane, known as β-CYD/CUR@MBN. This composite was designed to overcome the solubility and bioavailability issues inherent to CUR, a natural compound with potent anticancer properties but limited by poor water solubility and rapid degradation under physiological conditions [362,363,364]. The β-CYD/CUR inclusion complex prepared with different CUR:β-CD molar ratios (1:2, 1:4, and 1:6) demonstrated significantly enhanced stability and controlled release when embedded in the polymeric matrix. The sustained release profile of CUR, facilitated by the membrane structure, was validated through in vitro studies that demonstrated prolonged cytotoxic effects on GB cells. The β-CYD/CUR@MBN system preserved CUR’s integrity and functionality over time, thereby extending its therapeutic window and ensuring continuous anticancer activity. Notably, the membrane-based approach improved the localized delivery of CUR, reducing systemic exposure and minimizing potential side effects. This delivery system also exhibited selective cytotoxicity, remaining non-toxic to normal cells while effectively targeting tumor cells. Furthermore, the β-CD/CUR@MBN membrane reduced the cell availability of the two tested cell lines (Rat C6 GB and mouse B16F10 melanoma cell lines), while it was non-cytotoxic against normal cells.

Similarly, Mahdi et al. designed a multicomponent inclusion complex (CR-MC) of chrysin, a poorly soluble flavonoid with known anticancer properties [365,366], by combining it with HP-β-CYD and L-arginine (LA) through microwave irradiation. This CR-MC complex significantly enhanced chrysin’s solubility and dissolution, with a superior release profile (99.03 ± 0.39%) compared to the free compound (35.29 ± 1.55%). The formation of a stable structure was confirmed through spectroscopic (such as infrared spectroscopy, IR, or Nuclear Magnetic Resonance, NMR), DSC, and molecular docking studies, highlighting strong interactions between chrysin and HP-β-CYD essential for drug stability and bioavailability. SEM and DSC studies further revealed that the complex promoted an amorphous form of chrysin, contributing to its improved dissolution characteristics. In vitro tests on U87-MG GCM cells showed that CR-MC significantly enhanced the anticancer efficacy of free chrysin, reducing cell viability, increasing ROS production, and impairing mitochondrial membrane potential, thus inducing apoptosis and necrosis. Notably, the inclusion of L-arginine played a crucial role as an auxiliary agent, enhancing solubility and complexation efficiency by interacting with the CYD surface, aligning with prior findings that such agents can stabilize drug–CYD complexes and improve the solubility of poorly soluble drugs [367,368,369,370,371].

Last but not least, Colapietro et al. [372] highlighted the use of α-CYD in stabilizing sulforaphane (SFN), a natural compound derived from cruciferous vegetables with proven cytotoxic effects on glioma cells [373]. The α-CYD-SFN complex, known as SFX-01, demonstrated several benefits critical to GB treatment. Enhanced stability of SFN in the α-CYD formulation was a key advancement, addressing its rapid degradation and limited bioavailability. SFX-01 exhibited multifaceted mechanisms of action, including reducing GB cell growth, promoting differentiation, and inducing apoptosis via both intrinsic and extrinsic pathways. Preclinical models showed that SFX-01 effectively reduced tumor growth and improved survival rates, with potential synergistic effects when used alongside standard treatments. The study also identified molecular targets modulated by SFX-01, such as the downregulation of anti-apoptotic proteins (Bcl-2, Bcl-xL) and activation of pro-apoptotic proteins (Bax, Bad), as well as modulation of cell cycle regulators. Furthermore, SFX-01 demonstrated anti-angiogenic properties, crucial for limiting GB progression, and efficacy against GB stem cells, which play a significant role in treatment resistance and disease recurrence. Importantly, the α-CYD formulation provided an improved safety profile compared to free SFN, enhancing its potential for clinical application.

Table 2 provides a concise summary of the studies discussed in this section.

On this evidence, CYDs are emerging as versatile platforms for GB therapy, enabling improved solubility, stability, and bioavailability of challenging molecules. Their ability to form inclusion complexes supports innovative delivery strategies, including targeted and sustained release systems. By overcoming barriers such as poor water solubility and limited BBB and BBTB penetration, CYDs open new avenues for multimodal treatments and advanced formulations, positioning them as key players in next-generation oncology.

#### 8.7.2. CYDs in Liposomes

Liposomes are one of the most versatile drug delivery tools, owing to their unique bilayer structure that mimics cellular membranes, enabling the encapsulation of both hydrophilic and hydrophobic substances. This dual capability makes liposomes an ideal carrier for a broad range of therapeutic agents [244].

A detailed study by Lin et al. [374] demonstrated the synergistic use of liposomes with HP-β-CYD to enhance the delivery of butylidenephthalide (BP), a potent anticancer compound derived from *Angelica sinensis*. Despite its efficacy in inducing apoptosis in tumor cells, BP’s therapeutic application is hindered by its lipophilicity and chemical instability, which reduce its solubility, bioavailability, and overall effectiveness. The inclusion complex formed between HP-β-CYD and BP improved its solubility and stability, while liposomes provided additional protection and facilitated targeted delivery. The lipid bilayer of the liposomes stabilized BP and prolonged its systemic circulation, enhancing the drug’s likelihood of reaching target tissues. This characteristic is particularly critical in cancer therapy, as it reduces off-target effects and allows for controlled drug release. The BP-loaded liposomes were characterized by optimal particle size and encapsulation efficiency, ensuring effective drug delivery and interaction with biological barriers. Cytotoxicity assays confirmed that the formulation retained BP’s potent anticancer activity with increased efficacy compared to free BP. Fluorescence microscopy demonstrated successful internalization of the liposomes by tumor cells, confirming their role in targeted delivery. An especially innovative application involved a drug-resistant GB model. Intranasal administration enabled the formulation to bypass the BBB, delivering BP directly to brain tissues. LC-MS analysis confirmed BP localization within the tumor site, validating the system’s ability to overcome significant delivery challenges. This study underscores the synergistic potential of combining HP-β-CYD and liposomes in addressing the limitations of unstable or lipophilic drugs like BP. While HP-β-CYD enhances solubility and stability, liposomes offer additional protection and targeting capabilities, presenting a robust platform for advanced drug delivery.

Combining CYDs with liposomes creates a powerful synergy for drug delivery. CYDs improve the solubility and stability of lipophilic molecules, while liposomes provide protection, controlled release, and targeting capabilities. This integrated approach enhances therapeutic performance and enables innovative administration routes, positioning CYD–liposome systems as a promising platform for advanced cancer treatments, while reducing systemic toxicity.

#### 8.7.3. CYD-Based Nanocarriers

Recent advancements in CYD-based nanocarriers have highlighted their potential in self-assembly, enabling the efficient encapsulation and targeted delivery of hydrophobic therapeutic agents. Gallego-Yerga et al. [375] developed molecularly well-defined giant surfactants where β-CYD forms the hydrophilic moiety, coupled with a lipophilic calix[4]arene-based core. These surfactants self-assemble into NSs or NCs with an internal hydrophobic domain and an external β-CYD-based biocompatible shell. The β-CYD shell ensures aqueous compatibility, while the calix[4]arene core provides an optimal environment for encapsulating drugs such as TMZ, docetaxel (DTX), and combretastatin A-4 (CA-4). These nanoformulations achieved encapsulation efficiencies exceeding 80% and significantly improved drug solubility. Redox-sensitive designs, incorporating disulfide linkages between β-CYD and calix[4]arene, enabled controlled drug release in response to elevated glutathione levels in the TME. These systems exhibited remarkable cytotoxicity across various cancer cell lines, including prostate (LNCaP, PC3), GB (U87, C6), breast carcinoma (MCF-7), cervical carcinoma (HeLa), and colon carcinoma (HT-29). Encapsulation significantly enhanced apoptosis induction, reduced IC_50_ values, and amplified the drugs’ bioavailability and retention within cancer cells. Stability studies demonstrated enzymatic resistance and preservation under physiological conditions, ensuring minimal premature release and effective therapeutic delivery.

**Figure 12:**
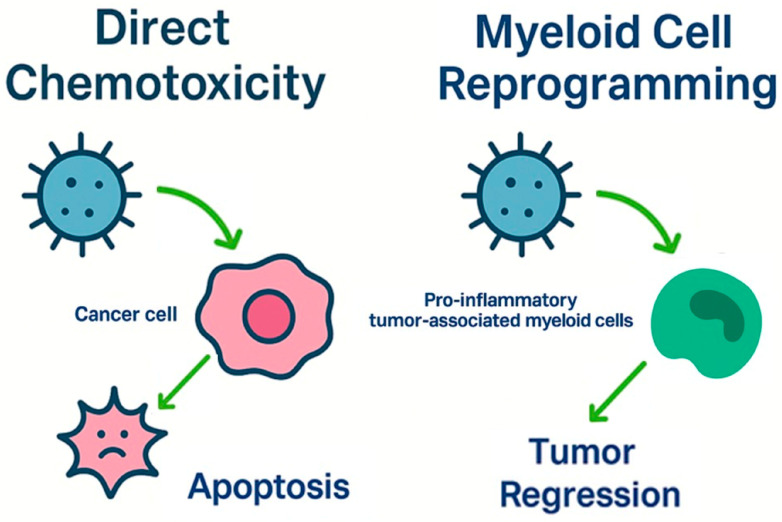
Schematic representation of the two mechanisms of action: direct chemotoxicity, where the drug acts directly on tumor cells, inducing cell death through cytotoxic effects; and myeloid cell reprogramming, where the drug modulates the tumor microenvironment by reprogramming myeloid cells, reducing their pro-tumoral activity and enhancing antitumor immune responses.

Two complementary therapeutic approaches are emerging in GB treatment, as illustrated in Figure 12: direct chemotoxicity, which targets tumor cells to induce cell death, and myeloid cell reprogramming, which modulates the tumor microenvironment to overcome immunosuppression, resulting in tumor regression.

Another notable study by Turco et al. [376] investigated β-CYD nanoparticles (CDNP) encapsulating the Toll-like receptor 7 and 8 (TLR7/8) agonist R848, targeting immunosuppressive tumor-associated myeloid cells (TAMs) in GB. CDNP-R848 induced significant antitumor effects, reshaping the immunosuppressive TME into a pro-inflammatory state. This transformation occurred independently of adaptive immune responses (T cells and NK cells), underscoring the critical role of myeloid cell reprogramming in glioma treatment. CDNP-R848 demonstrated selective uptake by GB-associated macrophages and monocyte-derived myeloid cells within the TME, bypassing significant challenges posed by the BBB. This targeted delivery was crucial for inducing a phenotypic shift in tumor-associated myeloid cells, characterized by the upregulation of pro-inflammatory markers such as F4/80 and MHC-II, along with enhanced production of ROS. At the same time, CDNP-R848 suppressed anti-inflammatory Ly6C+/PD-L1+ myeloid-derived suppressor cells (MDSCs), thereby mitigating the immunosuppressive conditions typically prevalent in GB. Mechanistic studies highlighted the role of TLR7/8 activation in reprogramming the myeloid compartment. CDNP-R848 induced a robust cytokine response within the TME, significantly upregulating IL-12, TNF-α, and other pro-inflammatory cytokines that play pivotal roles in orchestrating immune cell activation. Interestingly, while IL-12-mediated changes are typically associated with cytotoxic CD8+ T cell responses, the antitumor effects of CDNP-R848 were achieved independently of T cells and natural killer (NK) cells. Instead, the treatment relied predominantly on innate immune mechanisms driven by macrophages and their reprogrammed functionality. The therapeutic efficacy of CDNP-R848 was further demonstrated through its ability to induce substantial tumor regression in preclinical GB models. This was accompanied by prolonged survival rates, with some treated animals exhibiting complete tumor clearance. Importantly, CDNP-R848 achieved these outcomes with minimal systemic toxicity, a frequent limitation of free R848 administration due to widespread interferon-driven side effects. Advanced imaging techniques, including ultrasmall superparamagnetic iron oxide (USPIO) nanoparticle imaging, provided valuable insights into the dynamics of macrophage recruitment and infiltration during treatment. The imaging data revealed a decrease in macrophage influx in non-responder models, correlating with therapy resistance, while responders exhibited a significant reduction in tumor volume alongside a pro-inflammatory macrophage profile.

CYD-based nanocarriers represent a cutting-edge approach for targeted drug delivery. Their ability to self-assemble into stable, biocompatible structures enables high encapsulation efficiency, controlled release, and improved bioavailability of hydrophobic drugs. These systems can also integrate stimuli-responsive features and immunomodulatory functions, offering a versatile platform for precision oncology and next-generation GB therapies.

#### 8.7.4. CYD-based Systems for RNA Delivery

RNAi represents a highly conserved cellular mechanism that regulates gene expression through the degradation or translational repression of target mRNA molecules. This natural process has been strategically adapted to develop therapeutic approaches employing small interfering RNAs (siRNAs)—synthetic, double-stranded RNA molecules that precisely mimic the RNAi pathway to selectively silence pathogenic genes. Despite the success of siRNA-based therapeutics such as patisiran and givosiran, their clinical application is hindered by critical challenges, including poor stability, vulnerability to enzymatic degradation, and off-target effects [377,378]. To address these limitations, advanced delivery systems, particularly CYD-based NPs have emerged as promising non-viral vectors. β-CYD derivatives, in particular, exhibit biocompatibility, reduced immunogenicity, and robust capacity to shield nucleic acids until delivery to target cells, making them superior alternatives to viral vectors [379].

In this context, De La Torre et al. [380] developed AMC11, a β-CYD-based vector that forms supramolecular nanocomplexes with siRNA. These protect siRNA from RNase-mediated degradation and facilitate cellular uptake, enabling efficient gene silencing. AMC11 demonstrated exceptional transfection efficiency in multiple tumor cell lines, including GB (C6, U87) and prostate cancer (PC3, LnCaP) models. The nanocomplexes effectively silenced key regulatory proteins such as p42-MAPK and Rheb, which play critical roles in cell proliferation and survival, achieving more than 80% reduction in target protein expression. Remarkably, AMC11 exhibited low cytotoxicity, an essential criterion for clinical application.

In a parallel study, Manzanares et al. [381] investigated AMC6, another β-CYD-based derivative, designed to enhance siRNA delivery specifically through macropinocytosis. This pathway, prominent in tumor cells, involves the engulfment of extracellular fluid and its contents into large vesicles, enabling efficient internalization of siRNA nanocomplexes. AMC6-siRNA complexes displayed exceptional transfection efficiency in GB cell lines, reducing target protein levels such as CLTC, CAV1, and PAK1 by more than 80% and enabling detailed lack-of-function studies. Interestingly, while macropinocytosis was the predominant uptake route for AMC6, blocking this pathway did not entirely prevent siRNA internalization. Although macropinocytosis served as the primary uptake pathway for AMC6, the inhibition of this route did not completely block siRNA internalization, as alternative pathways, including clathrin-mediated and caveolin-mediated endocytosis, compensated to maintain high transfection efficiency. This adaptability highlights the robustness and versatility of AMC6 as a delivery vector.

CYD-based systems are emerging as efficient non-viral vectors for RNA delivery, offering protection against enzymatic degradation and enabling high transfection efficiency. Their biocompatibility and adaptability allow targeted uptake and controlled release, overcoming major barriers in gene silencing strategies. These features position CYDs as promising tools for advancing RNA therapeutics in oncology and beyond.

#### 8.7.5. CYDs as Diagnostic Contrast Agents

Contrast agents (CAs) are molecules extensively used to improve the diagnostic accuracy of magnetic resonance imaging (MRI). Traditional gadolinium-based CAs are widely used for their high relaxivity, but concerns about tissue retention and adverse effects in patients with impaired renal function have prompted the search for safer alternatives. Metal-free options such as nitroxide radicals (NRs) have emerged as promising candidates due to their enhanced biocompatibility and potential applications in areas like oxidative stress monitoring and tissue engineering. However, their clinical applicability remains limited by low longitudinal relaxivity and poor in vivo stability, particularly their susceptibility to reduction into non-paramagnetic hydroxylamines.

To overcome these challenges, Franco et al. [382] developed water-soluble polynitroxides derived from β-CYD, functionalized with nitroxides featuring either a piperidine (CD2, CD3) or pyrrolidine (CD4, CD5) structure. Advanced click-chemistry techniques enabled the attachment of nitroxides to the smaller rim of the β-CYD molecule while incorporating polar groups on the larger rim to enhance solubility. Pyrrolidine-based nitroxides (CD4, CD5) demonstrated superior stability under physiological reducing conditions due to their five-membered ring structure and steric hindrance from bulky substituents. These properties, confirmed through electron paramagnetic resonance spectroscopy and cyclic voltammetry, resulted in a significantly reduced rate of radical quenching compared to conventional mononitroxides.

The relaxivity (r1) of these polynitroxides was evaluated across magnetic field strengths relevant for clinical use (0.7 T to 9.4 T). CD4 and CD5 exhibited r1 values ranging from 0.6 to 1.9 s^−1^mM^−1^, with better performance at lower field strengths, reflecting the relaxivity–field dependence. Notably, CD4 displayed reduced sensitivity to field strength variations, a key advantage for clinical translation given that most MRI systems operate at 1.5–3 T. Cytocompatibility assays using human kidney (HEK293), mouse fibroblast (L929), and GB (U87) cells confirmed the non-toxic nature of these compounds at concentrations up to 1 μmol/mL, aligning with ISO 10993-12:2021 standards (Biological evaluation of medical devices—Part 12: Sample preparation and reference materials) [383]. Further validation came from in vivo MRI experiments on glioma-bearing rats, where CD3–CD5 were compared to gadolinium diethylenetriaminepentaacetate (Gd-DTPA). CD3 and CD4 provided prolonged tumor T1 relaxation and retained contrast for over 60 min, outperforming Gd-DTPA, which exhibited rapid signal decay. Pyrrolidine-based CD4 stood out due to its enhanced stability, higher tumor retention, and minimal off-target effects, while partial renal elimination was observed through bladder clearance. Despite favorable MRI performance, CD5 showed adverse effects in some animal models, highlighting the need for further safety evaluations.

Additionally, Yang et al. [384], developed an electrochemical immunosensor leveraging a graphene oxide (GO)-Fe_3_O_4_-β-CYD nanocomposite for detecting MGMT promoter methylation, a key biomarker in GB. This sensor uses anti-5-methylcytosine antibodies to specifically capture methylated DNA sequences. The integration of β-CYD enhanced sensitivity and enabled detection limits as low as 0.0825 pM. The platform demonstrated versatility, detecting both methylated DNA and other epigenetic modifications, such as N6-methyladenosine in RNA.

CYDs are gaining attention as innovative tools in diagnostic imaging and biosensing. Their structural versatility enables the development of safer, metal-free MRI contrast agents with improved stability and biocompatibility. Additionally, CYD-based nanocomposites enhance sensitivity in molecular diagnostics, supporting precise detection of critical biomarkers. These advances position CYDs at the intersection of imaging and personalized medicine.

## 9. Conclusions and Future Prospects

Glioblastoma (GB) remains one of the most challenging brain malignancies to treat, frequently exhibiting resistance to standard therapies. Achieving a favorable clinical outcome is exceedingly difficult due to its complex pathological features, robust resistance mechanisms, and the presence of two formidable barriers: the blood–brain barrier (BBB) and the blood–brain tumor barrier (BBTB). While the BBB restricts the passage of most therapeutic agents into the healthy brain parenchyma, the BBTB—formed through aberrant neovascularization in hypoxic tumor regions—adds an additional layer of complexity. Despite its increased permeability, the BBTB exhibits heterogeneous and selective transport properties, which can unpredictably hinder drug distribution within the TME. In this context, the development of innovative therapeutic strategies capable of overcoming both the BBB and BBTB is of paramount importance. CYD-based formulations represent a promising avenue in this field. CYDs possess a unique molecular architecture, characterized by a hydrophilic outer surface and a hydrophobic inner cavity, which enables them to interact with a wide range of biological macromolecules. This structural versatility underpins their growing relevance in pharmaceutical applications.

Several mechanisms contribute to the therapeutic potential of CYDs: inclusion complex formation with organic molecules enhances solubility in aqueous and biological media, improving drug bioavailability and absorption; membrane cholesterol modulation through complexation alters membrane permeability, facilitating increased uptake of active compounds; direct protein binding, enabled by specific amino acid configurations, supports targeted interactions and delivery; transmembrane transport, including across the BBB and potentially the BBTB, expands their utility CNS therapies.

Although further research is needed to optimize CYD-based drug delivery systems—particularly in terms of tumor specificity and formulation design—these compounds offer a non-toxic, biocompatible platform for encapsulating therapeutic agents and minimizing adverse effects.

However, clinical translation of CYD-based formulation remains a challenge. Formulation reproducibility, pharmacokinetic standardization, and long-term safety assessment represent important drawbacks to overcome.

To fully realize their potential, the future research on CYD-based formulations should have as its cornerstone the attempt to functionalize CYDs, obtaining advanced targeting strategies capable of recognizing tumor-specific receptors and crossing the blood–brain barrier. Combination of CYDs with polymeric or lipidic nanoparticles, could permit their employment in precision medicine, opening the possibility of tailoring CYD-based formulations to individual tumor profiles, maximizing treatment outcomes and minimizing recurrence. Overall, the next generation of CYD platforms should evolve toward targeted and patient-specific therapies capable of transforming GB management. CYDs stand at the frontier of advanced drug delivery systems.

## Figures and Tables

**Figure 8:**
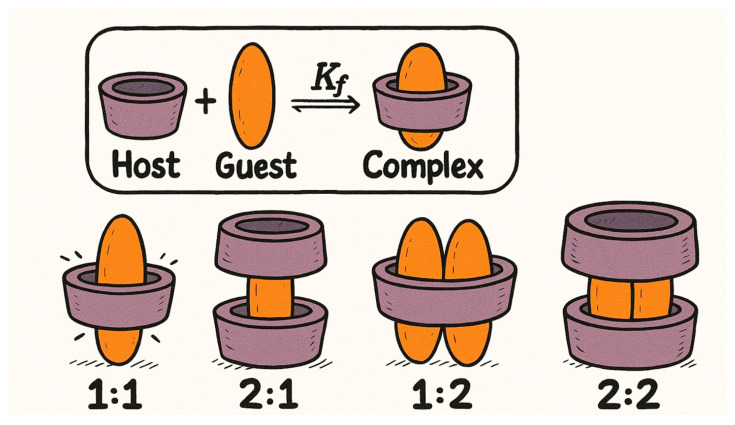
Schematic representation of host–guest interaction and different stoichiometric ratios (1:1; 2:1; 1:2; 2:2).

**Table 1:** Summary of treatment modalities with associated benefits and limitations.

*Treatment Modality*	Clinical Benefits	Limitations
* Surgical resection *	- Reduces tumor burden - Improves clinical symptoms - Enables identifying specific genetic mutations - Enhances efficacy of adjuvant therapies	- Often incomplete due to an infiltrative nature - Not feasible in eloquent brain areas - High recurrence rate near the surgical cavity
* Radiotherapy *	- Targets residual tumor cells - Extends survival - Palliative approach in inoperable patients to alleviate symptoms - Preserves surrounding healthy tissue	- Side effects (fatigue, headache, alopecia, cutaneous reactions, edema, cognitive decline) - Risk of radiation necrosis - Development of radio-resistance - Limited benefit from advanced techniques
* Pharmacotherapy *	- Prolong survival - Delay recurrence - Improve overall outcomes	- Poor infiltration of chemotherapy into tumor cells - Limited BBB and BBTB penetration - Resistance mechanisms reduce efficacy - Systemic toxicity (especially with IV agents) - Limited options after progression - Combination therapies are still under investigation
* Tyrosine Kinase Inhibitors (TKIs) *	- Target key molecular pathways - Potential for personalized therapy - New CNS-penetrant TKIs under development	- Limited BBB and BBTB penetration - Resistance mechanisms reduce efficacy - Modest clinical benefit - Significant side effects (e.g., hemorrhage, thrombosis)
* Tumor Treatment Fields (TTFs) *	- Selective and non-invasive - Improves survival when combined with TMZ - Minimal systemic toxicity	- High cost - Requires prolonged daily use - Hair shaving reduces patients’ compliance
* Supportive Therapy *	- Improves quality of life - Manages seizures, edema, pain, mood disorders, and cognitive decline - Reduces complications (e.g., VTE)	- Risk of side effects from long-term medications (e.g., corticosteroids, anticoagulants) - Requires individualized management - Limited efficacy

**Table 2:** Overview of inclusion complexes described in Section 8.7.1, including compounds, CYDs, molar ratios, key findings, applications, and references. Symbols: ↑ = increase; → = becomes/obtains; ↓ = decrease.

Compound	CYDs	Molar Ratio	Key Findings	Applications	Reference
* SuccFerr *	RAMEB	1:2	↑ solubility, ↑ cellular uptake, ↑ apoptosis, ↑ drug availability, potent antiproliferative effect	GB therapy (in vitro U87/F98; in vivo rat models)	[349]
* Cannabidiol (CBD) *	HP-β-CYD, RAMEB, DM-β-CYD, TM-β-CYD	1:37 1:5 1:1 1:1	cytotoxic effect dose- and time-dependent, ↑ solubility, ↑ stability, ↑ bioavailability	GB and RMS therapy (in vitro A172 and TE-671)	[353]
* Disulfiram * * (DSF) *	HP-β-CYD	Not specified	↑ solubility (~2450-fold), combined with Cu → ↑ antitumor activity, bypass BBB via intranasal delivery	GB therapy (in vitro C6, in vivo rat models)	[354]
* Temozolomide (TMZ) *	β-CYDamine, βCYDgal, β-CYD	1:1	↑ solubility, ↑ stability, ↑ bioavailability potential to overcome BBB and improve uptake	GB therapy	[358]
* Photosensitizers *	β-CYD	2:1	↑ solubility, ↓ aggregation, ↑ cellular uptake; stable complexes	GB therapy and photodynamic treatment (in vitro U251 and U87*)*	[361]
* Curcumin * * (CUR) *	β-CYD (embedded in cationic starch/PVA membrane)	1:2 1:4 1:6	↑ stability, controlled release, prolonged cytotoxicity on GB cells, selective toxicity, membrane-based localized delivery	GB therapy (in vitro C6 and B16F10)	[364]
* Chrysin *	HP-β-CYD + L-arginine	Not specified	↑ solubility, ↑ dissolution, release profile 99%, ↑ anticancer efficacy (ROS ↑, apoptosis ↑)	GB therapy (in vitro U87-MG cells)	[366]
* Sulforaphane (SFN) *	α-CYD (SFX-01)	Not specified	↑ stability, ↓ degradation multifaceted anticancer effects (apoptosis, anti-angiogenic, GB stem cell targeting), improved safety profile	GB therapy (preclinical models)	[372]

## Data Availability

No new data were created or analyzed in this study. Data sharing is not applicable to this article.

## Abbreviations

The following abbreviations are used in this manuscript:

PDGFR-α	Platelet-derived growth factor receptor-α
CA	Contrast agent
saRNA	Self-amplifying mRNA
5-ALA	5-aminolevulinic acid
AED	Antiepileptic medication
APC	Antigen-presenting cell
ASCO	American Society of Clinical Oncology
BBB	Blood–Brain Barrier
BBTB	Blood–Brain Tumor Barrier
BP	Butylidenephthalide
CA-4	Combretastatin A-4
CAR-T	Chimeric Antigen Receptor T-cell
CBD	Cannabidiol
CBM	Cannabis-based medicine
CBT	Cognitive–behavioral therapy
CDKN2A/B	Cyclin-dependent kinase inhibitor 2A/B
CED	Convection-enhanced delivery
CLE	Confocal laser endomicroscopy
CMV	Cytomegalovirus
CNS	Central nervous system
CUR	Curcumin
CYD	Cyclodextrin
DAMP	Damage-associated molecular pattern
DDSs	Drug delivery systems
DKD	Diabetic kidney disease
DM-β-CYD	2,6-O-methyl-β-CYD
DOAC	Oral anticoagulant
DSC	Differential scanning calorimetry
DSF	Investigating disulfiram
DTX	Docetaxel
EGFR	Epidermal growth factor receptor
EGFRvIII	Epidermal Growth Factor Receptor Variant III
EMA	European Medicines Agency
EORTC/NCI	European Organization for Research and Treatment of Cancer/National Cancer Institute
Cmax	High concentration peak
EPR	Permeability and retention
FDA	Food and Drug Administration
FGS	Fluorescence-guided surgery
FTIR	Fourier-transform infrared spectroscopy
GB	Glioblastoma
G-CIMP	Hypermethylated phenotype
GRAS	Generally Recognized as Safe
HER2	Human Epidermal Growth Factor receptor 2
HPLC	High-performance liquid chromatography
HP-β-CD	hydroxypropyl-β-cyclodextrin
ICH	Intracranial hemorrhage
IDH	Isocitrate dehydrogenase
IL-13Ra2	Interleukin 13Rα2 Receptor
iMRI	Intraoperative Magnetic Resonance Imaging
IR	Infrared spectroscopy
iUS	Intraoperative ultrasound
LA	L-arginine
LMWH	Low-molecular-weight heparin
MRI	Magnetic resonance imaging
mRNA	Messenger RNA
MTIC	5-(3-methyl-1-triazen-1-yl)imidazole-4-carboxamide
MTT	3-(4,5-dimethylthiazol-2-yl)-2,5-diphenyltetrazolium bromide
NC	Nanocapsule
NEC	Not Elsewhere Classified
Nf1	Neurofibromatosis type 1
NK	Natural killer ì cell
NMR	Nuclear Magnetic Resonance
NOS	Not Otherwise Specified
NS	Nanospheres
NSAID	Non-steroidal anti-inflammatory drug
NSCLC	Non-small cell lung cancer
OS	Overall survival
OV	Oncolytic virus
PAMPs	Pathogen-associated molecular patterns
PCV	Procarbazine, lomustine, and vincristine
PDGFRA	Platelet-Derived Growth Factor Receptor Alpha
PDT	Photodynamic therapy
PES	Progression-free survival
PFS	Progression-free survival
PTEN	Phosphatase and tensin homolog
RADIONECROSIS	Radiation-induced tissue damage
RAMEB	Randomly methylated β-CYD
RBC	Red blood cell RBC
RMS r	Rhabdomyosarcoma
RNAi	RNA interference
ROS	Reactive oxygen species
RTCT	Radiotherapy and chemotherapy
SBE-β-CYD	Sulfobutyl ether β-cyclodextrin
SEM	Scanning electron microscopy
SF	Sodium fluorescein
SFN	Stabilizing sulforaphane
SNRI	Serotonin-norepinephrine reuptake inhibitor
SSRI	Selective serotonin reuptake inhibitor
SuccFerr	N-[4-ferrocenyl,5-5-bis(4-hydroxyphenyl)-pent-4-enyl]-succinimide
TAA	Tumor-associated antigen
TCA	Tricyclic antidepressant
TERT	Telomerase reverse transcriptase
TKs	Tyrosine kinases
TKIis	Tyrosine kinase inhibitors
TMZ	Temozolomide
TM-β-CYD	2,3,6-tri-O-methyl-β-CYD
TNF	Tumor Necrosis Factor
TP53	Tumor protein p53
TTFs	Tumor Treatment Fields
USPIO	Ultrasmall superparamagnetic iron oxide
VEGF	Vascular endothelial growth factor
VTE	Venous thromboembolism
WHO	World Health Organization
XRD	X-ray diffraction
β-CYDamine	6-monodeoxy-6-monoamino-β-CYD hydrochloride
βCYDgal	β-CYD derivatives with galactosamine and triazole rings
